# A systematic review of specialized psychosocial and complex psychosocial interventions for early psychosis, early depression, early bipolar disorder, and early borderline personality disorder

**DOI:** 10.1192/j.eurpsy.2026.10158

**Published:** 2026-03-06

**Authors:** Andreas Bechdolf, Hendrik Müller, Daniel Richter, Stefan Weinmann, Thomas Becker, Steffi G. Riedel-Heller, Uta Gühne

**Affiliations:** 1 Department of Psychiatry, Psychotherapy, and Psychosomatics, Vivantes Klinikum Am Urban and Vivantes Klinikum im Friedrichshain, Berlin, Germany; 2 Department of Psychiatry and Psychotherapy, Charité – Universitätsmedizin Berlin, Berlin, Germany; 3 German Center for Mental Health (DZPG), Berlin, Germany; 4 Department of Psychiatry and Psychotherapy, Faculty of Medicine and University Hospital Cologne, Köln, Germany; 5 Department of Psychiatry, Psychotherapy, and Psychosomatics, Vivantes Klinikum Am Urban, Berlin, Germany; 6 Institute of Social Medicine, Occupational Medicine and Public Health (ISAP), University of Leipzig, Leipzig, Germany; 7 Institut für Qualitätssicherung und Transparenz im Gesundheitswesen (IQTIG), Berlin, Germany; 8 Zentrum für Integrative Psychiatrie, Universitätsklinikum Schleswig-Holstein, Lübeck, Germany; 9 Department of Psychiatry and Psychotherapy, University of Leipzig Medical Center, Leipzig, Germany

**Keywords:** complex interventions, early intervention, early severe mental disorders, psychosocial interventions, systematic review

## Abstract

**Background:**

This systematic review evaluates specialized psychosocial and complex interventions for early bipolar disorder (BD), early borderline personality disorder (BPD), early depression, early psychosis, and first-episode mental illness in general (FEMI).

**Methods:**

We included systematic reviews and randomized controlled trials (RCTs) of interventions with psychosocial components, excluding trials that focused on pharmacological-only interventions and stand-alone psychotherapies. Searches were conducted in January 2023 across five databases. Review quality was assessed using AMSTAR-2 and risk of bias for RCTs using the Cochrane tool.

**Results:**

Ten studies met the inclusion criteria: seven reviews and three RCTs. High-to moderate-quality evidence supports complex psychosocial interventions combined with pharmacotherapy for early psychosis. The most robust effects were reductions in relapse and improvements in psychosocial functioning; additional benefits were observed for symptom burden, remission, treatment discontinuation, and hospital admissions. Benefits were most sustained in longer-duration, community-based programs. For early BD, limited evidence suggests that combining pharmacotherapy with family-focused therapy or structured psychoeducation may improve the course of illness and treatment satisfaction. One RCT in early BPD reported improved engagement with a developmentally tailored program. Two FEMI RCTs found that nurse-led psychoeducation and psychosocial programs improved in-patient duration, symptoms, insight, self-efficacy, quality of life, and engagement. No eligible studies addressed early-stage depression, indicating a notable evidence gap for multimodal psychosocial interventions.

**Conclusions:**

Complex psychosocial interventions are strongly supported for early psychosis. Preliminary data in BD, BPD, and FEMI suggest consistent benefits for engagement, but further rigorous trials – especially in early depression – focusing on different outcomes – are required.

## Introduction

Most severe mental illnesses (SMI) emerge during adolescence and young adulthood (63–75% by age 25) [1, 2]. The biological, cognitive, and social changes that occur during youth and adolescence create vulnerabilities to mental illness during these periods. Young people with psychosis, bipolar disorder, depression, or borderline personality disorder face an increased risk of developing persistent symptoms, reduced functioning, and a significantly heightened risk of developing SMI [1, 3, 4]. The unique challenges are best addressed by interventions tailored to this age group [1]. Thus, specialized psychosocial interventions and complex psychosocial interventions have been developed to promote recovery in young people at substantial risk of developing SMI [5, 6]. Specialized phase-specific services typically provide a multidisciplinary team that meets the needs of young individuals in the initial or early stages of illness. These complex psychosocial interventions aim to improve clinical, functional, and vocational outcomes and reduce barriers to accessing care through a coordinated and integrated combination of psychotherapy, psychoeducation, support with education, vocational therapy, and psychopharmacology interventions [5].

The efficacy of specialized psychosocial and complex interventions provided in early intervention services compared to control intervention or standard treatment has primarily been examined in young people with early psychosis. Consequently, numerous reviews and meta-analyses have assessed these strategies in this population, demonstrating advantages over treatment as usual across various outcomes [5–7].

A comprehensive review of the efficacy of specialized psychosocial and complex interventions in other mental disorders, such as early depression, early bipolar disorder, and early borderline personality disorder, is currently missing. This review should draw on evidence from reviews, meta-analyses, and controlled trials, similar to early psychosis, as these disorders also carry a risk of developing SMI [3, 4]. Therefore, there is a need for an in-depth exploration of the evidence for specialized psychosocial and complex interventions in mental disorders beyond psychosis.

This review aims to assess the evidence for multi-professional collaborative specialized psychosocial and complex interventions, including psychoeducation and support in education and employment, whether provided together or as individual interventions alongside psychopharmacotherapy or psychotherapy for early depression, early bipolar disorder, and early borderline personality disorder. Additionally, we aim to present an updated review of the evidence regarding specialized psychosocial and complex interventions in early psychosis.

## Methods

This review was prepared as part of the second revision of the evidence- and consensus-based guideline “Psychosocial therapies for severe mental illness” developed under the leadership of the German Society of Psychiatry and Psychotherapy, Psychosomatics and Neurology (DGPPN). The guideline, which identifies adults with SMI as the target group, defines SMI as a duration of illness of more than 2 years with significant impairments to psychosocial functioning [8].

This systematic review was conducted according to Preferred Reporting Items for Systematic Reviews and Meta-Analyses (PRISMA) guidelines [9]. A predetermined, unpublished protocol was followed (Supplementary Material 1).

### Inclusion and exclusion criteria

The studies included in this systematic review met the stated PICO criteria: (1) Population: Patients experiencing their first episode of a mental illness at high risk for SMI within 5 years of the first diagnosis. Age: early intervention studies often focus on children and adolescents. For this review, we established the age criterion to include only those studies where at least most participants were over 18 years old; (2) Intervention: Psychosocial interventions were defined following the S3 guideline “Psychosocial therapies for severe mental illness” (DGPPN e. V. (Hrsg.) für die Leitliniengruppe 2019) [10] and included complex interventions with psychosocial components and circumscribed psychosocial interventions (e.g., psychoeducation, peer support, lifestyle intervention); (3) Comparison: other active interventions, or standard care; (4) Outcomes: a) classic clinical and patient-related outcome indicators (relapses, in-patient readmissions, psychiatric symptom severity, treatment continuity, utilization behavior), and b) treatment satisfaction and recovery-oriented indicators (e.g., empowerment, quality of life, inclusion in work, psychosocial functioning).

In an initial step, all reviews and meta-analyses that met the search criteria were included. In a second step, individual randomized controlled trials (RCTs) were considered for diagnostic groups for which no reviews or meta-analyses were available.

### Search strategy

A systematic literature search was conducted on January 12, 2023 (with no limits applied for year of publication). It included five databases: The Cochrane Database of Systematic Reviews, The Cochrane Central Register of Controlled Trials, Pubmed, PsychInfo, and Embase. A manual review of reference lists from eligible publications and relevant review articles supplemented this search. In addition, studies known to the experts on the guideline committee were taken into account.

Search terms included keywords and MeSH terms and were arranged in groups; *Group 1* for population: severe mental illness, severe psychiatric disorder, severe mental health problems, depression, depressive disorder, severe affective disorder, schizophrenia, psychosis, bipolar disorder, bipolar affective disorder, manic and depression, personality disorder, borderline personality disorder, AND *Group 2* for stage: early-phase, early-stage, first-episode, first-presentation, AND *Group 3* for interventions: early psychosocial intervention, early intervention, early treatment, early intervention or service or program, early psychosocial treatment, psychosocial intervention, integrated treatment, first episode program* or service, early onset team, AND *Group 4* for study design: randomized controlled trial, random allocation, random trial or study, random allocation, systematic review or search, meta-analysis, and scoping review.

This review summarizes the Group 3 search terms under “specialized psychosocial and complex interventions.”

More information on the search strategy for each database can be found in Supplementary Material 1.

Following the previously described eligibility criteria, two reviewers screened each title and abstract independently (DR, UG); any differences in inclusion/exclusion determinations were discussed before proceeding with the review. Two reviewers (DR, UG) conducted every full-text screening, discussing and resolving differences.

### Data extraction

Two review authors (DR, UG) extracted data from the included publications and compared the data extraction results. Any discrepancies were discussed.

### Risk of bias and quality assessment

For the included reviews, two review authors (DR, UG) applied the AMSTAR 2 checklist. The AMSTAR 2 checklist is a tool for critically appraising systematic reviews of healthcare interventions [11]. The raters assess the quality of a systematic review or meta-analysis using 16 items. When evaluating the overall certainty of the results of the included systematic reviews, we defined seven items as critical items based on the recommendations of the AMSTAR author group (questions 1, 4, 7, 9, 11, 13, 15).

Two reviewers (DR, UG) evaluated each RCT for risk of bias using the Cochrane Risk of Bias Tool (RoB 1) for Randomized Controlled Trials [12]. This tool comprises seven categories of potential bias, and each category was assessed as having a low, high, or unclear risk of bias.

We examined the identified review articles for duplicates to ensure that RCTs were not overrepresented by being included in multiple reviews (Supplementary Table 4).

### Qualitative synthesis

We described findings relating to the patient groups identified, including systematic reviews and RCTs. To enhance readability, the results section only presents the outcomes with consistently positive effects of the complex psychosocial interventions across reviews. These outcomes were: general symptoms, remission, psychosocial functioning, relapse rates, in-patient admissions, duration of in-patient admissions, and treatment discontinuation. All other outcomes are reported in Supplementary Material 1.

## Results

**Figure 1.**
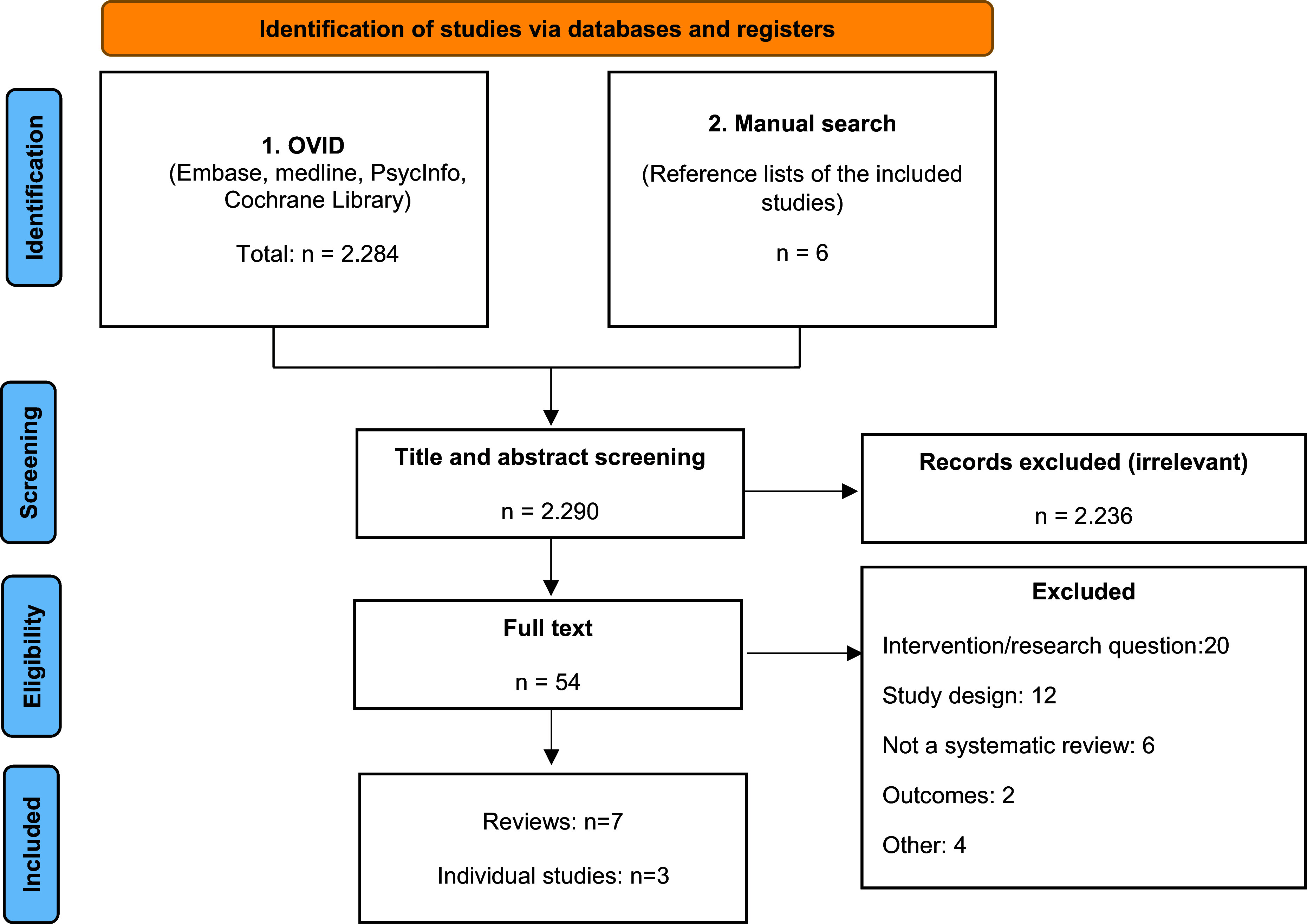
PRISMA flow-chart of included publications.

The systematic literature search identified seven review articles (six systematic reviews with meta-analyses [5, 7, 13–16] and one systematic review [17]) and three RCTs [18–20] that met the inclusion criteria. The PRISMA flow chart (Figure 1) illustrates the number of studies included and excluded at each stage.

The results of the systematic reviews and meta-analyses will be presented first, followed by the individual studies.

The meta-analyses included participants with a median age range of 20 to 28.6 years and sample sizes between 1145 and 2811. The studies with the largest number of participants came from Australia and Great Britain (six each), followed by Hong Kong (five), Denmark and the USA (four each), Spain (three), China, Mexico, and the Netherlands (two each), and Italy, Norway, and Sweden (one each).

Most data for specialized psychosocial and complex interventions are currently available for people with early psychosis [5, 7, 13–16]. Only one recent review was identified that included people with bipolar disorder in the early course of illness [17]. Most reviews examined specialized psychosocial and complex interventions or combinations of several evidence-based approaches consisting of psychosocial therapies and psychotherapy in the experimental interventions combined with psychopharmacological interventions in both the experimental and the control condition. Thus, isolating the effects of individual elements or combinations is challenging. Where possible, the focus is directed towards psychosocial approaches. All basic characteristics of the reviews are given in Table 1.

**Table 1.** Overview of basic characteristics of the aggregated evidence on the effectiveness of coordinated care in people within the early course of disorders with increased risk of severe mental disorders

Authors (Year)	Number of includedStudies/study designCountry of study	Patients Diagnosis(s) (Mean) age in years Number of participants (min, max.) Mean duration of the disorder Mean duration until first treatment	Experimental intervention Type of intervention Duration of intervention/study (min, max) Intensity	Control inventory	Length Follow-ups
Ratheesh et al. (2023) [17] Systematic Review	Extraction of 5 RCTs: Kessing et al. (2013) [21], Miklowitz et al. (2003) [22], Miklowitz et al. (2008) [23], Miklowitz et al. (2014) [24], Colom et al. (2010)[25] *n.*s.	Persons with a bipolar disorder (early phase) 34 to 35 years (*k* = 3), 14 to 15 years (*k* = 2) *N* = 58 to 158	**Psychotherapy and psychosocial approaches**: Family-oriented psychoeducation (*k* = 3), structured group psychoeducation (*k* = 1), specialized multimodal outpatient care (*k* = 1) *n*/a *n*/a	Conventional therapy (*k* = 1) psychopharmacological therapy alone (*k* = 1), non-structured group intervention (*k* = 1), enhanced care (*k* = 1), crisis intervention (*k* = 1)	*n*/a
Frawley et al. (2021) [13] Systematic review with meta-analysis	31 RCTs *n.*s.	People with early experiences of psychosis 22.3 (SD 3.6) years, *k* = 4 with patients under 18 years) *n* = 2.811 (27, 392) K = 11 ultra-high-risk Participants (*n* = 1,040), *k* = 2 Pat. in prodromal-stage (*n* = 126) *k* = 11 patients with psychosis in the first episode (FEP) (*n*= 1,171), *k* = 7 early psychosispsychosis up to 5 yearsafter diagnosis) (*n* = 474)	**Psychotherapy and** **Psychosocial approaches**: CBT (cognitive behavioral therapy; *k* = 8), FBT (family-based therapy; *k* = 3), Supported employment (*k* = 3), CRT (cognitive remediation therapy; *k* = 10), multimodal psychosocial interventions (*k* = 7) 2 months to 3 years (mean = 8.7 [SD = 7.7]) Number of sessions: 9 to 128 (mean = 32.1 [SD = 24.2])	Treatment as usual (*n* = 9), multimodal psychosocial intervention (*n* = 8), supportive therapies (*n* = 5), case management (*n* = 3),	*n*/a
Puntis et al. (2020) [7] Systematic review with meta-analysis	4 RCTs Great Britain (*n* = 1), Denmark *n* = 1), Sweden (*n* = 1), USA (*n* = 1)	People with a first episode of psychosis (within 3 years, max. 2 episodes) 23–27 years *n* = 1.145 (range 50–547) *n*/a up to 24 months untreated disorder or max. 6 months Psychopharmacotherapy	**Multimodal** treatment concepts through specialized **early intervention services** (including medication management, family intervention, resilience training, Supported Employment, problem-solving training, crisis management, CBT, symptom management, social skills training 18–24 months *n*/a.	treatment as usual (*n* = 4)	up to 10 years
Camacho-Gomez and Castellvi (2020) [14] Systematic review with meta-analysis	11 RCTs (14 papers) 11 Australia (*k* = 1), China (*k* = 1), Denmark (*k* = 1), Great Britain (*k* = 1), Hong Kong (*k* = 4), Netherlands (*k* = 1), Spain (*k* = 2)	People with a first episode of psychosis N = 1.360 16.1 to 28.1 years	**Family interventions** Manualized family interventions (psychoeducation, communication training, problem-solving approaches, *k* = 5), multi-component treatment: psychoeducation, mutual support, problem-solving approaches, social skills training, CBT, family group session (*k* = 5), mutual support group (*k* = 1) 6 to 18 months	TAU (*k* = 7), TAU supplemented by by elements such as education, training for recognition of early warning signs (*k* = 1), Community Mental Health Teams (*k* = 1), unstructured group sessions (*k* = 1), EPPIC program (*k* = 1)	6 to 24 months
Correll et al. (2018) [5] Systematic review with meta-analysis	10 RCTs (42 papers) Great Britain (*n* = 2), Mexico (*n* = 2), USA (*n* = 2), Italy (*n* = 1), Norway (*n* = 1), Denmark (*n* = 1), Hong Kong (*n* = 1)	People with a first episode of psychosis or in an early phase of schizophrenia spectrum disorder 27,5 (23–37) *N* = 2.176 (range: 50–547) 159.8 (125.4) weeks (6 studies) 79.9 (71.1) weeks (5 studies)	**Specialized** **intervention services (EIS)**: team-based multimodal treatment concepts (e.g. psychopharmacotherapy, psychoeducation, vocational support, crisis management, family counseling/therapy) 9–24 months (average: 16.2 (7.4) months)	treatment as usual (*n* = 10)	*n*/a
Marshall and Rathbone (2011) [15] Systematic review with meta-analysis	12 RCTs Australia (*k* = 5); Spain (*k* = 1), Great Britain (*k* = 2); Netherlands (*k* = 1); Denmark (*k* = 1); USA (*k* = 1); China (*k* = 1)	People with a first episode of psychosis No systematic data (20 to 26 years) *n* = 24, 547 *n.*a. *n.*a.	**Psychotherapy and** **Psychosocial approaches**: (cognitive) behavioral therapy (*k* = 2), family therapy (*k* = 2), Integrated therapy (Assertive Community Treatment, family therapy, social skills training social skills training) (*k* = 1), LEO-CAT (*k* = 1; Lambeth Early Onset Crisis Assessment Team), IPS (*k* = 1), Psychoeducation (*k* = 1), psychopharmacotherapy alone (*k* = 1), VT + cannabis + psychosis therapy (*k* = 1), other (*n* = 2) 2–24 months *n*/a	Treatment as usual (*n* = 6), treatment as usual + supportive therapy (*n* = 1), placebo (*n* = 1), psychoeducation (*n* = 1), other (*n* = 3)	*n*/a
Alvarez-Jimenez et al. (2011) [16] Systematic review with meta-analysis	9 RCTs *n*/a	People with first episode of psychosis 20–29 years *n* = 1.411 (33, 547)	**Psychotherapy and** **Psychosocial approaches**: special early intervention programs *k* = 3), individual cognitive behavioral therapy (*k* = 3), combined individual and family-oriented cognitive behavioral therapy with a focus on relapse prevention (*k* = 1), Family-oriented therapy (*k* = 1) 5–12 weeks *k.* A.	(multidisciplinary) Treatment as usual (*k* = 5), case management (*k* = 3), other (*k* = 1)	7–24 months

Based on the AMSTAR 2 ratings, the quality of evidence for systematic reviews and meta-analyses was graded as high [5, 15], moderate [7, 14, 16, 17], or low [13]. The details on the AMSTAR-2 assessment are given in Supplementary Table 1.

### Evidence from meta-analysis and systematic reviews

#### Specialized psychosocial and complex interventions *in the treatment of people with early psychosis*

In a specialized early intervention service, a phase-specific, multidisciplinary team provides treatment to individuals in the early stages of a (psychotic) illness or those experiencing first-episode psychosis. This team offers a range of treatments, including psychopharmacotherapy, psychotherapy, psychoeducation, family intervention, support in education and employment, and other psychosocial therapies. Therefore, the services described in this section are considered comprehensive early intervention services.

In a recent Cochrane review, the effectiveness of specialized early intervention teams (SEI) was compared to TAU for individuals with first-episode psychosis [7]. Two studies reported on recovery from psychosis at the end of treatment, indicating that care by the SEI team led to a higher rate of recovery (defined here as a global state) compared to TAU (73% vs. 52%; RR = 1.41 [95% CI: 1.01–1.97]; *p* = 0.04; *k* = 2, *n* = 194; *I* ^2^ = 18%; low certainty). No apparent differences were observed during the follow-up period up to 60 months after the end of treatment. Effects were evident for lower service non-utilization or dropout risk with SEI (RR = 0.50 [95% CI: 0.31–0.79]; *p* = 0.003; *k* = 3; *n* = 630; *I* ^2^ = 0%; moderate certainty). There was low-certainty evidence that SEI at the end of treatment may lead to fewer admissions to psychiatric hospitals than TAU (52% vs. 57%; RR = 0.91 [95% CI: 0.82–1.00]; *p* = 0.04; *k* = 4, *n* = 1,145), and low-certainty evidence that SEI may be associated with fewer psychiatric hospital days (MD -27.00 days [95% CI: −53.68 to −0.32]; *p* = 0.04; *k* = 1; *n* = 547). Long-term follow-up (more than 60 months after treatment) showed no significant differences. Regarding psychosocial functioning, a significant difference in favor of SEI was observed at the end of treatment (SMD 0.37 [95% CI: 0.07–0.66]; *p* = 0.01; *k* = 2; *n* = 467 participants; *I* ^2^ = 45%; low-certainty evidence), however, not during the follow-up period. There was no effect on employment status (RR = 1.21 [95% CI: 0.94–1.55]; *p* = 0.15; *k* = 2; *n* = 951). Moreover, no substantial differences were observed regarding overall symptom severity at the end of treatment, risk of relapse, quality of life, or mortality. Significantly fewer SEI patients discontinued studies prematurely (RR = 0.63 [95% CI: 0.54–0.74]; *p* < 0.00001; *k* = 4; *n* = 1,145; *I* ^2^ = 0%; moderate certainty).

An earlier Cochrane Review by Marshall and Rathbone (2011) included psychosocial interventions among the evaluated approaches. The review also considered studies involving cognitive behavioral therapy, pharmacological treatments, and specialized early intervention teams. This broad scope led to significant heterogeneity among the studies, which were generally characterized by small sample sizes and numerous methodological limitations. As a result, meta-analyses were typically not feasible. Consequently, no reliable conclusions could be drawn regarding the effectiveness of the included psychosocial interventions [15].

In a meta-analysis conducted by Correll et al. (2018), encompassing 10 RCTs with a total of 2,176 participants, a comparative analysis was undertaken between early intervention services (EIS) and TAU in individuals with early psychosis (Correll et al., 2018). The risk of treatment discontinuation could be reduced (21.3% vs. 31.3%; RR = 1: 0.70 [95% CI: 0.61–0.80]; *p* < .001; *k* = 10), the risk of psychiatric hospitalizations was reduced (at least one admission, RR = 0.74 [95% CI: 0.61–0.90] *p* = .003; *k* = 10), and overall symptom severity (SMD: −0.32 [95% CI: −0.47 to −0.17]; *p* < .001; *k* = 8). EIS also reduced the risk of relapse (RR = 0.71 [95% CI: 0.53–0.93]; *p* = .01, *k* = 7;), and promoted recovery, defined as symptom stability or minimal severity plus improved social, academic, and vocational development (RR = 1.24 [95% CI: 1.03–1.5], *p* = .02; *k* = 3), a positive effect on psychosocial functioning was also observed (SMD: 0.21 [95% CI: 0.09–0.34]; *p* = .001; *k* = 7). The superiority of EIS was consistent across almost all treatment time points (6, 9–12, and 18–24 months) [5].

Overall, the mean effect sizes are considered small for continuous variables; effect sizes for categorical outcomes are considered small to moderate. More improvement compared to the TAU group was observed in other outcomes, such as remission (NNT = 5.7), relapse prevention (NNT = 10.0), hospitalization (NNT = 10.1), and treatment adherence (NNT = 12.4). Subgroup analyses were conducted; however, the small number of participants limited conclusions. Differences in adherence scales favored EIS (risk reduction by fidelity scales RR = 0.88 vs. 0.50; *p* = .001). Participation in education and employment and a better global functional level seemed to be associated primarily with vocational and educational counseling and family interventions.

A previous review showed that specialized early intervention programs are more effective in preventing relapse than standard treatment approaches. (OR = 1.80, 95% CI: 1.31–2.48; *p* < .001; NNT = 10; *N* = 679; *k* = 3) [16]. These programs provided a comprehensive range of specialized and phase-oriented out-patient and in-patient treatments, including outreach services (antipsychotics, manualized CBT strategies, individual crisis management plans, family counseling, and psychoeducation). TAU was standard care provided by non-specialized psychiatric healthcare providers. There was no evidence of statistical heterogeneity (*I* ^2^ = 0; *p* = .82). Additionally, a statistically significant reduction in average days of in-patient treatment was observed for participants in the early intervention programs compared to those receiving usual treatment (weighted mean difference = 26.20 days [95% CI: 7.35–45.06 d; *p* < .01; *I* ^2^ = 0%, *p* = .71). A combined relapse prevention approach (individual and family-based) was marginally superior to SEI alone ((*k* = 1; OR = 0.21 [95% CI: 0.04–1.03], *p* = .06, *k* = 1; *N* = 81).

#### Early intervention in individuals with first-episode psychosis through specific psychosocial approaches

The following section considers those studies that focus on **specific (early) interventions with a psychosocial focus.**

The publication by Frawley et al. (2021) is relevant to this review as it explicitly sought studies examining changes in social and occupational functioning during the early stages of psychotic disorders [13]. Social and occupational functioning, or participation, were assessed using the following criteria: (1) global functioning, measured by standardized instruments [e.g., Global Assessment of Functioning (GAF)], and (2) individual definitions of functioning covering one or more of the following areas: vocational participation, educational participation, level of independence, and social participation. Improvements in psychosocial functioning were observed across all interventions studied (SMD = 0.239 [95% CI: 0.115–0.364], *p* < 0.001). Effect sizes varied based on intervention type, stage of illness, treatment length, duration, and outcome measurements used. Multi-component interventions were associated with the most significant improvements [13].

Camacho-Gomez and Castellvi (2020) investigated the effectiveness of family interventions for up to 24 months of follow-up in preventing relapses and other relapse-related outcomes in patients with first-episode psychosis [14]. Pooled results showed that compared to TAU and/or other psychosocial interventions, family interventions were effective in preventing relapses during follow-up (RR = 0.42 [95% CI: 0.29–0.61], *p* < 0.000001; *k* = 6, *I* ^2^ = 1%). Family interventions also proved to be effective compared to TAU alone (RR = 0.36 [95% CI: 0.21–0.63], *p* = 0.0003; *I* ^2^ = 9%; *k* = 4) and TAU plus other psychosocial measures (RR = 0.48 [95% CI: 0.27–0.86], *p* = 0.01; *k* = 2, *I* ^2^ = 14%). Family interventions also showed benefits in reducing the duration of hospital stay (TAU: MD = -3.31 days [95% CI:-6.48 to −0.14], *I* ^2^ = 71%; other interventions: MD = -4.57 days [95% CI: −7.49 to −1.65]; *k* = 8), and increased psychosocial functioning level (TAU: SMD = 1.36 [95% CI: 0.59–2.12; *I* ^2^ = 94%; other interventions: SMD = 1.41, 95% CI: 0.87–1.96; *k* = 6).

#### Specialized and complex psychosocial interventions for people with early bipolar disorder

In a recent review, Ratheesh et al. (2023) investigated interventions for individuals with bipolar disorder in the early stages of the illness [17]. Family-focused therapy (FFT) was examined alongside psychopharmacological treatment in three RCTs, one of which demonstrated an extension of time to relapse: 73.5 weeks ±28.8 compared to 53.2 weeks ±39.6 in the comparator group (enhanced care, crisis management, or briefer family intervention) [26]. However, definitive conclusions regarding the effectiveness of FFT in preventing the recurrence of episodes in individuals with bipolar disorder in the early stage cannot be drawn. Two of the studies included adolescent patients. In another study (*N* = 158) identified by Ratheesh’s review, the effectiveness of specialized multi-modal out-patient care (guideline-conforming pharmacological interventions and group-based psychoeducation post-discharge) versus TAU (routine out-patient psychiatric services) was investigated [21]. The risk of later readmission was significantly lower among those receiving the specialized intervention (HR = 0.60, 95% CI: 0.37–0.97). Structured group psychoeducation, compared to supportive group intervention, led to a significant extension of time to relapse among participants with ≤6 prior episodes (log-rank 4.3, *p* = 0.04) but not among participants with >6 prior episodes [25] (Table 2).

**Table 2.** Effects of early interventions in people within early course of disorders with increased risk of severe mental disorders on various outcomes in systematic reviews/meta-analyses

	Frawley et al., 2021 [13]		Puntis et al., 2020 [7]	Camacho-Gomez and Castellvi, 2020 [14]	Corell et al., 2018 [5]	Alvarez-Jimenez et al., 2011 [16]		
Experimental vs. control intervention	MCPI vs. CON	FBT vs. CON	EIS vs. TAU		EIS vs. TAU	Spec. FEP-program vs. TAU	CBT + Spec. FEP-program vs. Spec. FEP-program alone	
**Number of studies (*k*)**	*k* = 7	*k* = 3	***k* = 4**	*k* = 11	***k* = 10**	*k* = 3	*k* = 2	
Quality of life			**~**		**++**			
Participation in education/work			**~**		**++**			
Recovery					**++**			
Remission			**++**		**++**			
Symptoms (total)			**~**		**++**			
Symptoms (positive)					**++**			
Symptoms (negative)					**++**			
Symptoms (general)				**++**	**++**			
Depression					**++**			
Relapse rate			~	**++**	**++**	**++**	**~**	
Number of in-patient admissions			**++**		**++**			
Duration of in-patient admissions			**++**	**++**	**++**	**++**		
Psycho-social-functioning	**++**	**~**	**++**	**++**	**++**			
Treatment satisfaction			**++**					
Study dropout			**++**					
Treatment discontinuation			**++**		**++**			

*Note:* ++: Significant advantage in experimental group compared to control group, +: tendential superiority without significant difference in experimental group compared to control group, ~: Results comparable in both groups, −: disadvantage in experimental group compared to control group.

Marshall and Rathbone (2011) [15] was not included in the overview, as no reliable statements on psychosocial interventions are possible based on the analysis. Results based on *k* = 1 studies were excluded from this table.

Abbreviations: EIS, early intervention service; FBT, Family-Based Therapy; FEP, first episode psychosis; MCPI, multi-component psychosocial intervention; TAU, treatment as usual.

### Evidence from individual studies

This systematic review additionally identified three individual studies. One involved young adults with early borderline personality disorder [18], while the other two examined participants presented with early symptoms of SMI [19, 20] (Table 3). These three RCTs were included because they addressed diagnostic populations not covered by the identified reviews and meta-analyses.

**Table 3.** Characteristics of individual studies on the effectiveness of specialized psychosocial and complex psychosocial interventions for early psychosis, early depression, early bipolar disorders, and early borderline personality disorders

Author	Patient population	Intervention	Control group
Year Country of study	Number (N) Diagnosis Average age in years (SD) Duration of illness Setting	Approach Modules/content Duration/follow-up	
**Chanen et al. 2022 [18]** Australia	N=139 Borderline personality disorder according to the Structured Clinical Interview for DSM-IV-TR Axis II Disorders. 19 (2.8) N/A Comprehensive community psychiatric treatment.	**Specific treatment model** **Treatment arm 1: Helping Young People Early (HYPE) + cognitive analytic therapy (CAT)** - integrative clinical case management, general and acute acute psychiatric care, collaborative approach, self-confident commitment, outreach care in the community, functional recovery, active involvement of the family (HYPE) - time-limited psychotherapy (self-management; CAT) - **Treatment arm 2: HYPE + Befriending** - Exchange on interests such as e.g., sports, music, or other topics, no illness-related exchange (Befriending) - Duration 18 months	TAU adapted for younger persons (YMHS) + Befriending - Multidisciplinary approach
**Chien et al.,** 2015 [20]	• N=180 First-onset psychotic and mood disorders within the past three months, newly referred to mental healthcare services, and presenting at least mild to moderate–severe levels of psychiatric symptoms (i.e., Brief Psychiatric Rating Scale score of >25 out of 126 and/or Chinese version of the Beck Depression Inventory-II scores of >10 out of 63), but with no history of and low risk of suicide and self-harm. • 25 (8.3) • 3 months • Outpatient care in general psychiatric outpatient clinics.	Coping with the disease and possible symptoms: (1) Orientation, involvement, (2) cooperation and motivational interviewing; (3) Social skills training; (4) Improvement of coping strategies; (5) Individual Review and development of perspectives. - Duration 4 months (8 two-hour sessions every two weeks); months follow-up	Treatment as usual in outpatient psychiatric routine services: Medical advice consultation and usual treatment by a psychiatrist and referrals to psychosocial and social services (nursing, social work)
**Chien & Leung** 2013 [19]	N=96 First moderate symptoms of the first episode of mental illness, as assessed by the Brief Psychiatric Rating Scale (25–48/127) and Beck’s Depression Inventory II (10–29/63). 26 (6.6) 1.4 months Outpatient care in general psychiatric outpatient clinics.	Needs-oriented psychoeducation program: (1) Orientation, engagement and understanding of mental health health/disorder; (2) Sleep hygiene and anxiety relief; (3) treatment and medication; (4) coping; (5) interpersonal skills; (6) community resources and perspectives - Duration 1h sessions every 2 weeks over 3 months; follow-up: 6 months.	- Treatment as usual in outpatient psychiatric routine services: Counselling and treatment planning by a psychiatrist, counselling via community services, brief information of the family about mental illness by specialist care or social worker.

Age ranged from 19 to 26 years. The sample size varied from 96 to 180. Studies were conducted in Australia (*k* = 1) and Hong Kong (*k* = 2). The interventions evaluated included psychoeducation [19, 20] and combined approaches [18]. All studies were conducted in out-patient or community mental health settings and included multi-component treatments.

Study quality: We graded all eligible trials (3/3, 100%) as low risk of bias in sequence generation [18–20]. The methods used for sequence generation were computer-generated sequencing or drawing a labeled card. All trials (3/3, 100%) had an unclear risk of bias for allocation concealment. Team-based and psychosocial interventions are complex care interventions and would be difficult to mask. Therefore, none of the three trials blinded participants and the treatment staff from the treatment arm allocation. We graded all three eligible studies as high risk of bias for blinding. We graded all studies at low risk of bias for blinding of outcome assessments. We graded three eligible trials (3/3, 100%) as low risk of bias concerning incomplete outcome data and for selective reporting. We did not think there was a high risk of other potential sources of bias within the included trials.

Chanen et al. (2022) evaluated the efficacy of three early intervention strategies for individuals with borderline personality disorder (BPD) in the early stages of illness. In this study, participants were either (1) provided with helping young people early (HYPE), a specialized BPD treatment model for young people, combined with weekly cognitive analytic therapy (CAT); (2) given HYPE in combination with weekly befriending psychotherapy as a control condition; or (3) offered a general youth psychiatric treatment model (YMHS), combined with befriending. Planned comparisons (YMHS + Befriending vs. HYPE; HYPE + CAT vs. Befriending) indicated that neither the care model nor the psychotherapeutic intervention was associated with a higher rate of change in psychosocial functioning up to the primary 12-month endpoint. HYPE outperformed YMHS + Befriending in terms of treatment engagement (median [IQR], 22 [19] vs. 3 [16] contacts; median duration, 200 [139.5] vs. 94 [125] days) and treatment completion (44 out of 92 [47.8%] vs. 9 out of 47 [19.2%]). HYPE + CAT surpassed Befriending in treatment engagement (median [IQR], 12 [16.5] vs. 3 [9.8] sessions) and treatment completion (24 out of 46 [52.2%] vs. 29 out of 93 [31.2%]). Early intervention for BPD in this study did not appear contingent on specialized psychotherapy [18].

Chien and Bressington (2015) examined a nurse-led structured psychosocial intervention program incorporating psychoeducational elements implemented in a psychiatric out-patient setting for people with a first episode of mental disorders [20]. These results showed significant positive effects compared to treatment-as-usual on psychiatric symptomatology (primary outcome; *p* = 0.001; ES = 0.56), insight into disorder, and quality of life during a 12-month follow-up period. Additionally, the program reduced the duration of recurrent hospitalizations compared to TAU.

Similarly, Chien and Leung (2013) evaluated a needs-based psychoeducation program led by specialized nursing staff for outpatients experiencing their first episode of a mental disorder (Tables 3 and 4) [19]. The findings demonstrated significant improvements in mental health outcomes compared to standard outpatient care (primary outcome; need-based psychoeducation: mean 3.0 vs. control: mean 4.8; *p* < 0.001), insight into disorder and need for treatment, self-efficacy, and duration of hospitalization over a 6-month follow-up period.

**Table 4.** Effects on specialized psychosocial and complex psychosocial interventions for early psychosis, and early borderline personality disorders on various outcomes in single trials

Author/year	Chanen et al., 2022 [18]		Chien & Leung, 2013 [19]	Chien & Bressington, 2015 [20]
Treatment arms	HYPE + CAT vs. HYPE + Befriending	HYPE vs. YMHS + Befriending	Psychoeducation vs. TAU alone	Psycho-social-intervention program vs. TAU alone
Quality of life				++
Self-efficacy			++	~
Symptoms (general)			++	++
Insight (disorder; treatment)			++	++
Depression	~	~		
Borderline personality disorder severity Index	~	~		
Substance abuse	~	~		
Suicide Ideation	~	~		
Relapse rate		~		
Duration of in-patient admissions			++	++
Psycho-social-functioning	~	~		
Treatment satisfaction	~	~		
Treatment discontinuation	++	++		

Notes: ++: A significant advantage in the experimental group compared to the control group, +: a tendency towards superiority without a significant difference in the experimental group compared to the control group, ~: Results are comparable in both groups, -: a disadvantage in the experimental group compared to the control group.

CAT: Cognitive Analytic Therapy; CSC: Coordinated Specialty Care; HYPE: Helping Young People Early; I-CAT: Integrated Coping Awareness Therapy; YMHS: Youth Mental Health Service.

## Discussion

This systematic review aimed to evaluate specialized psychosocial and complex interventions for early psychosis, early bipolar disorder, early depression, and early borderline personality disorder. The systematic literature search identified seven review articles – six systematic reviews with meta-analyses and one systematic review – that met the inclusion criteria. Participants’ ages ranged from 20 to 28.6 years. The mostly high to moderate quality of evidence from reviews, meta-analyses, and trials allows for the drawing of reliable conclusions across different domains.

### Early psychosis

**Figure 2.**
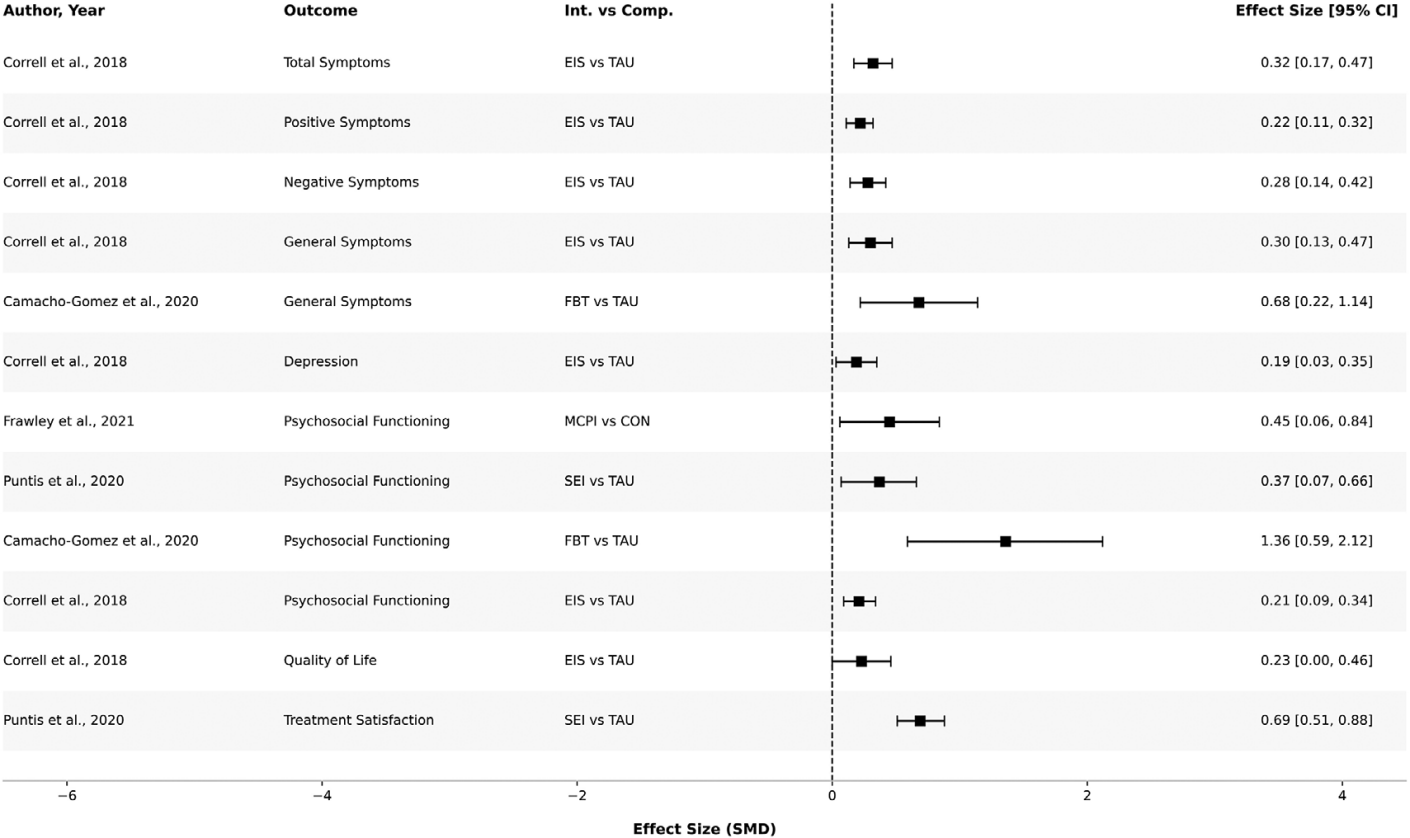
Effect sizes of interventions on symptoms, psychosocial functioning, and subjective outcomes early psychosis. *Note*: Con, control group; EIS, early intervention services; FBT, family-based therapy; MCPI, multi-component psychosocial interventions; TAU, treatment as usual; SEI, specialized early intervention services. For improved interpretability, symptom reductions are presented as positive effect sizes in the forest plot. Due to the transformation of the standard error, the values of the 95% confidence intervals may differ slightly. Effect sizes with 95% confidence intervals that included null were excluded from the figure.

**Figure 3.**
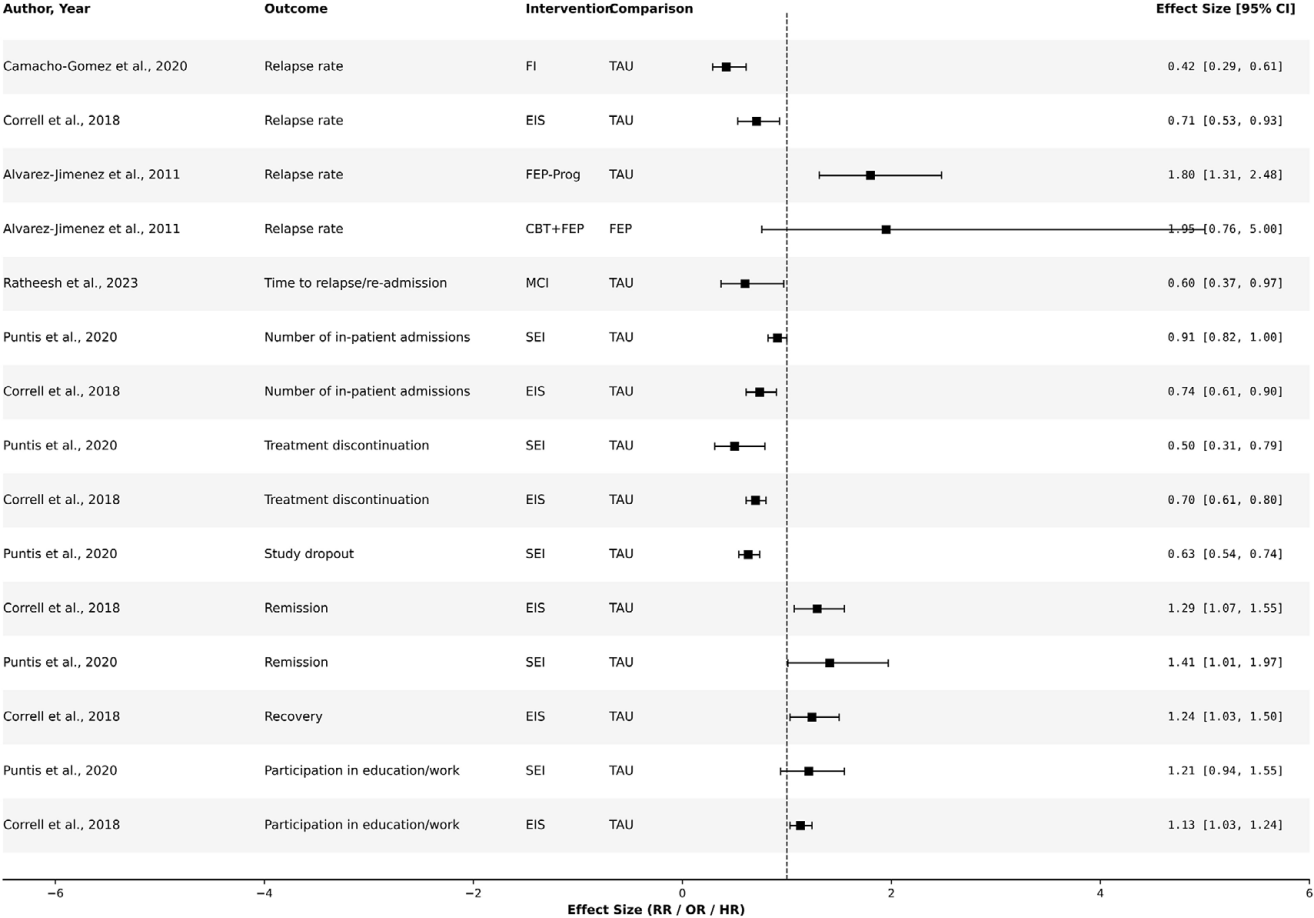
Effect sizes of binary endpoints in early psychosis and early bipolar disorders. *Note*: CBT, cognitive behavioral therapy; CI, crisis Intervention; EIS, early intervention services; FBT, family-based therapy; FEP-P, specialized first episode program; FFT, family-focused therapy; RP, relapse prevention; TAU, treatment as usual; SEI, specialized early intervention services. Due to the transformation of the standard error, the values of the 95% confidence intervals may differ slightly. Effect sizes with 95% confidence intervals that included the null were excluded from the figure.

For individuals with early psychosis, multiple meta-analyses and systematic reviews consistently demonstrate the superiority of specialized, multicomponent early intervention services over treatment as usual. These complex interventions typically incorporate pharmacotherapy alongside psychosocial therapies such as cognitive behavioral therapy, psychoeducation, family intervention, supported employment, social training, and cognitive remediation. Continuous outcomes, such as psychosocial functioning and overall symptom severity, consistently demonstrate significant improvements, typically within the small to moderate range (Figure 2). Compared to individual interventions, complex approaches yielded the strongest functional improvements [13]. Strong effects of early intervention in psychosis were also observed for dichotomous outcomes, particularly reductions in relapse and hospitalization rates (Figure 3). Moreover, coordinated assertive outreach and community-based care have been shown to improve treatment continuity and decrease service disengagement in individuals with early-stage psychosis [5, 7, 13]. Taken together, these results indicate that multimodal psychosocial interventions in early psychosis not only prevent deterioration of acute illness but also promote functional recovery and improvements in daily life, and engagement with services, thereby targeting both clinical stability and broader recovery-oriented outcomes. These benefits are reliably observed across different reviews and treatment durations, with interventions lasting longer than 6 months being particularly effective [13].

### Early bipolar disorder

For early bipolar disorder, the evidence base is more limited. Still, it indicates that combining pharmacotherapy with family-focused therapy or group-based psychoeducation within specialized mood disorder services can enhance illness management and treatment satisfaction [17]. Although these results are promising, more rigorous trials are needed to confirm the findings and their applicability to other outcomes.

### Early borderline personality disorder

In early borderline personality disorder, a single trial suggests that developmentally targeted, integrated interventions may improve treatment engagement and adherence compared to standard care. While improvements in clinical symptomatology have not consistently reached statistical significance, the authors suggest that enhanced engagement represents an intermediate outcome that may help with long-term recovery [18].

### Early depression

This review did not identify reviews or RCTs evaluating specialized psychosocial interventions for early depression, highlighting a notable gap in the literature. Importantly, this absence of evidence does not imply that interventions in the early stages of depression lack empirical support. Recent meta-analyses have examined the efficacy of psychotherapy alone [28, 29] or in combination with antidepressant medication [30] in early depression. However, these meta-analyses focused exclusively on children and adolescents and/or did not meet the inclusion criteria for complex psychosocial interventions of this review [28–30].

### The first episode of a mental disorder in general

For first-episode mental disorders across diagnoses, nurse-led psychoeducation and psychosocial programs significantly improved the duration of in-patient admissions, general symptoms, insight, self-efficacy, and quality of life compared to standard care [19, 20]. These interventions target newly referred patients who would otherwise face waiting times before their first specialist consultation. By offering immediate, needs-based, community-focused care, these programs were explicitly designed to fill gaps in community mental health care and provide low-threshold access to multimodal psychosocial interventions, leading to high rates of engagement and treatment completion.

### Limitations

A methodological limitation of this review is the potential bias arising from the repeated inclusion of individual trials across meta-analyses and systematic reviews. However, the comparison (Supplementary Material 2, Table 4) showed that the overlap was relatively low, with the OPUS I trial appearing in three reviews [14–16] and the RAISE trial in two reviews [5, 13]. Additionally, this review focused on adult populations (see age range), which may have influenced the results and conclusions. Including younger participants in this review might have yielded different findings.

In conclusion, the main findings of this review show that for individuals with early psychosis, complex psychosocial interventions provide consistent benefits for relapse prevention, symptom reduction, treatment engagement, and across various functional domains. These effects may be due to the following mechanisms: 1. Multimodal frameworks – characterized by interdisciplinary collaboration and structured service delivery, ensure that complex individualized health needs are addressed [5]. 2. Complex psychosocial interventions are particularly effective when delivered through assertive outreach and community-based models, as these offer easy access to care, ensure treatment continuity, and prevent disengagement from services [5, 7, 13]. Furthermore, complex psychosocial interventions may themselves enhance engagement with services and treatment satisfaction, as supported by the findings in early borderline personality disorder, early bipolar disorder, and first-episode mental disorders in general [17, 18], indicating the transdiagnostic potential of these interventions. The findings on first-episode mental disorders in general show that low-threshold, needs-based interventions can effectively bridge service gaps and achieve high engagement, further emphasizing their transdiagnostic potential [19, 20]. 3. Improvements in social and occupational functioning during early psychosis may result from multimodal services that include components such as vocational and educational support, as well as family-based therapy [5, 13, 14], since these interventions actively involve patients and their social environment in real-world settings.

**Figure 4.**
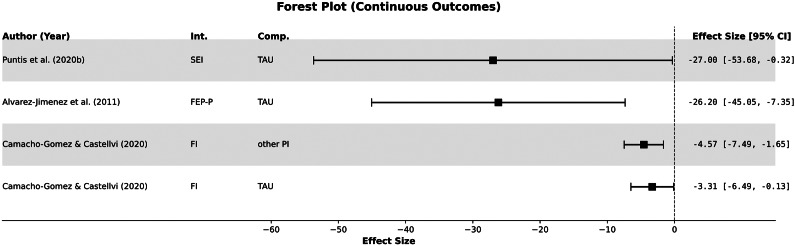
Reduction of inpatient admission in days in early psychosis. *Note*: FEP-P, specialized first episode program; FI, family Intervention; PI, psychosocial Intervention; TAU, treatment as usual; SEI, specialized early intervention services. Due to the transformation of the standard error, the values of the 95% confidence intervals may differ slightly. Effect sizes with 95% confidence intervals that included the null were excluded from the figure.

This review demonstrates that individuals experiencing early psychosis episodes benefit from timely, coordinated, and comprehensive care delivered by specialized multidisciplinary teams. Such services should integrate pharmacotherapy with evidence-based psychosocial interventions – including cognitive behavioral therapy, family interventions, psychoeducation, and vocational or educational support – delivered flexibly across service stages and settings, ideally within assertive outreach and community-based models. Multicomponent frameworks show promise for reducing relapse or inpatient admissions, and preventing further social and functional deterioration, thereby supporting both clinical stabilization and broader recovery-oriented outcomes (Figure 4).

Preliminary findings in bipolar disorder, borderline personality disorder, and first-episode mental disorders in general suggest that such complex psychosocial approaches may also enhance service engagement beyond psychosis. This preliminary evidence suggests a potential transdiagnostic value of multimodal, recovery-oriented interventions.

For all conditions beyond psychosis – particularly early depression – more high-quality research is needed to assess the effects of complex psychosocial interventions across a broader range of outcomes.

## Supporting information

10.1192/j.eurpsy.2026.10158.sm001 Bechdolf et al. supplementary material 1 Bechdolf et al. supplementary material

10.1192/j.eurpsy.2026.10158.sm002 Bechdolf et al. supplementary material 2 Bechdolf et al. supplementary material

## Supplementary material

The supplementary material for this article can be found at http://doi.org/10.1192/j.eurpsy.2026.10158.

## Artificial intelligence

AI-assisted tools were employed solely for language refinement and stylistic editing during manuscript preparation.
