# Supplementary material for: A systematic review of specialized psychosocial and complex psychosocial interventions for early psychosis, early depression, early bipolar disorder, and early borderline personality disorder
Source: Eur Psychiatry. 2026 Mar 6;69(1):e47. doi: 10.1192/j.eurpsy.2026.10158 (PMC13122531; doi:10.1192/j.eurpsy.2026.10158)
Supplement: Bechdolf et al. supplementary material 1 — Bechdolf et al. supplementary material [file S0924933826101588sup001.docx]

**A systematic review of specialized psychosocial and complex psychosocial Interventions for** **early psychosis, early depression, early bipolar disorders, and early borderline personality disorders**

**Supplement 1: Study Protocol**

**Review Question (s)**

This review aims to determine whether evidence exists to support multi-professional, collaborative, and specialized psychosocial and complex interventions—including psychoeducation and support in education and employment—as standalone approaches or in combination with psychopharmacotherapy or psychotherapy for individuals in the early stages of depression, bipolar disorder, and borderline personality disorder. Furthermore, it seeks to provide an updated synthesis of the current evidence regarding the effectiveness of specialized psychosocial and complex interventions in early psychosis.

In short: What evidence supports the effectiveness of multi-professional, collaborative, specialized psychosocial, and complex interventions for early depression, early bipolar disorder, early borderline personality disorder, and early psychosis?

**Searches**

Databases:

The following databases will be searched to identify systematic reviews of randomized controlled trials and randomized controlled trials:

EBM Reviews - Cochrane Central Register of Controlled Trials

EBM Reviews - Cochrane Database of Systematic Reviews

Embase (1974 to present)

Ovid MEDLINE(R) ALL (1946 to present)

APA PsycInfo (1806 to present)

Search Terms:

In order to identify relevant studies, our search strategies will use a combination of subject headings and free text search terms, including the following terms:

Group 1 for population: severe mental illness, severe psychiatric disorder, severe mental health problems, depression, depressive disorder, severe affective disorder, schizophrenia, psychosis, bipolar disorder, bipolar affective disorder, manic and depression, personality disorder, borderline personality disorder, AND Group 2 for stage: early-phase, early-stage, first-episode, first-presentation, AND Group 3 for interventions: early psychosocial intervention, early intervention, early treatment, early intervention or service or program, Early Psychosocial treatment, psychosocial intervention, integrated treatment, First episode program* or service, Early onset team, AND Group 4 for study design: randomized controlled trial, random allocation, random trial or study, random allocation, systematic review or search, meta-analysis, scoping review.

The search strategy will be developed by the review team in accordance with the clinically relevant research questions within the framework of the S3 guidelines for psychosocial therapies for severe mental illnesses and their cross-diagnostic focus. There are no restrictions to publication period. Papers in English and German will be considered.

**Types of study to be included**

Inclusion: (1) Patients experiencing their first episode of a mental illness at high risk for SMDs within 5 years of the first diagnosis. Age: Early intervention studies often focus on children and adolescents; for this review, we set the age criterion to include only studies in which at least the majority of participants are over 18 years old. (2) Systematic reviews of randomized controlled trials and randomized controlled trials (RCTs). (3) Interventions: Psychosocial interventions defined in accordance with the S3 guideline "Psychosocial therapies for severe mental illness." (DGPPN e. V. (Hrsg.) für die Leitliniengruppe 2019) [10] and including complex interventions with psychosocial components or circumscribed psychosocial interventions (e.g., psychoeducation, peer support, lifestyle intervention); (4) Comparison: other active interventions, wait list, or standard care.

Exclusion: (1) Population: The majority of the patients examined are under 18 years of age. (2) Intervention: lack of focus on psychosocial approaches.

**Condition or Domain to be studied**

Psychosocial interventions are defined according to the S3 guideline "Psychosocial therapies for severe mental illness." (DGPPN e. V. (Hrsg.) für die Leitliniengruppe 2019) [10] and including complex interventions with psychosocial components or circumscribed psychosocial interventions (e.g., psychoeducation, peer support, lifestyle intervention) in individuals experiencing their first episode of a mental illness at high risk for SMDs within 5 years of the first diagnosis.

**Participants**

Inclusion criteria:

(1) Adolescents and adults aged >/=18 years old more than 50 percent

(2) Study-defined diagnosis of first-episode psychosis or early-phase schizophrenia-spectrum disorder, or another early diagnosis with an increased risk of severe progression (such as depression, major depressive disorder, major affective disorder, bipolar disorder, bipolar affective disorder, manic depression, personality disorder, borderline personality disorder)

**Intervention(s), Exposure(s)**

This review will include any treatment consisting of psychosocial interventions defined in accordance with the S3 guideline "Psychosocial therapies for severe mental illness." (DGPPN e. V. (Hrsg.) für die Leitliniengruppe 2019) [10] and including complex interventions with psychosocial components or circumscribed psychosocial interventions (e.g., psychoeducation, peer support, lifestyle intervention, supported employment, family intervention).

**Comparator(s)/Control**

The control will be those who did not receive early psychosocial intervention for the early phase of mental illness.

**Outcomes**

We will use the following outcomes:

(1) Classic clinical and patient-related outcome indicators (relapses, inpatient readmissions, psychiatric symptom severity, treatment continuity, utilization behavior), and

(2) treatment satisfaction and recovery-oriented indicators (e.g., empowerment, quality of life, inclusion in work, psychosocial functions).

**Data Extraction (selecting and coding)**

Study selection:

Citations and available abstracts of the search results will be uploaded in Excel and screened for potential eligibility. This process will occur in two stages. The first stage will involve screening titles and abstracts to exclude studies that do not meet the inclusion criteria. Two reviewers will independently screen all citations. Discrepancies will be resolved through consensus, and if an agreement cannot be reached, a third reviewer will be involved. In the second stage, at least two reviewers will independently screen the full text of the remaining studies and assess them for eligibility.

For studies excluded during this stage, a reason for exclusion will be recorded for later reporting. Any discrepancies at this stage will be resolved by consulting a third reviewer, who will independently assess the study. For studies with substantial overlap, the most informative review will be included.

Data extraction:

Data will be extracted using a template for the entire guideline. Information will be extracted on the following:

1. study identification (title, authors, and year of publication, country),
2. sample characteristics (sample size, age, diagnosis, duration of illness),
3. study characteristics (design, nature of intervention, control intervention, topics, length and intensity of intervention, and length of follow-up),
4. and main findings.

**Assessment of methodological quality of systematic reviews**

AMSTAR 2 is used to evaluate the methodological quality of systematic reviews. All of the 16 items are first evaluated individually. The ratings of the AMSTAR 2 items are then used to derive an overall confidence rating for the results of the systematic reviews. Seven of the 16 items (questions 1, 4, 7, 9, 11, 13, 15) are considered “critical items”. Depending on how many critical and non-critical questions are answered with ‘No’ (or not with ‘Yes’ or ‘Partly Yes’), the overall confidence in the results of the review will be rated as ‘high’, ‘moderate’, ‘low’, or ‘critically low’.

Two reviewers will independently assess the quality. Discrepancies will be resolved through consensus.

**Risk of bias (quality) assessment: Individual studies (RCTs)**

Each included study (RCT) will be assessed using the Cochrane Risk of Bias (RoB 2) tool, a validated instrument designed to assess the quality of randomised trials. Two reviewers will independently assess study quality and generate a ROB score. Discrepancies will be resolved through consensus.

**Strategy for Data Synthesis**

Narrative/qualitative Synthesis

**Type and method of review**

Narrative review

**Language**

English

**Country**

Deutschland

**Keywords**

Early intervention, Psychosocial interventions, Severe mental illness, Youth mental health, Integrated care

**Contact details for further information**

Andreas Bechdolf, Department of Psychiatry, Psychotherapy, and Psychosomatics, Vivantes Klinikum Am Urban and Vivantes Klinikum im Friedrichshain, Berlin, Germany.

E-Mail: andreas.bechdolf@vivantes.de

**Review Team**

Andres Bechdolf (Department of Psychiatry, Psychotherapy, and Psychosomatics, Vivantes Klinikum Am Urban and Vivantes Klinikum im Friedrichshain, Berlin, Germany, Department of Psychiatry and Psychotherapy, Charité – Universitätsmedizin Berlin, Berlin, Germany, German Center for Mental Health (DZPG), Berlin-Potsdam site, Germany), Hendrik Müller (Department of Psychiatry and Psychotherapy, Faculty of Medicine and University Hospital Cologne, Cologne, Germany), Daniel Richter (Institute of Social Medicine, Occupational Medicine and Public Health (ISAP), University of Leipzig, Faculty of Medicine, Leipzig, Germany, Institut für Qualitätssicherung und Transparenz im Gesundheitswesen (IQTIG), Berlin, Germany), Stefan Weinmann (Zentrum für Integrative Psychiatrie, Universitätsklinikum Schleswig-Holstein, Lübeck, Germany), Thomas Becker (Department of Psychiatry and Psychotherapy, University of Leipzig, Leipzig, Germany), Steffi G. Riedel-Heller (Institute of Social Medicine, Occupational Medicine and Public Health (ISAP), University of Leipzig, Faculty of Medicine, Leipzig, Germany), Uta Gühne (Institute of Social Medicine, Occupational Medicine and Public Health (ISAP), University of Leipzig, Faculty of Medicine, Leipzig, Germany)

**Funding sources**

The revision of the evidence- and consensus-based guideline "Psychosocial therapies for severe mental illness” and the systematic searches is supported by the German Association for Psychiatry, Psychotherapy and Psychosomatics (DGPPN).
