# Supplementary material for: A systematic review of specialized psychosocial and complex psychosocial interventions for early psychosis, early depression, early bipolar disorder, and early borderline personality disorder
Source: Eur Psychiatry. 2026 Mar 6;69(1):e47. doi: 10.1192/j.eurpsy.2026.10158 (PMC13122531; doi:10.1192/j.eurpsy.2026.10158)
Supplement: Bechdolf et al. supplementary material 2 — Bechdolf et al. supplementary material [file S0924933826101588sup002.docx]

**A systematic review of specialized psychosocial and complex psychosocial Interventions for** **early psychosis, early depression, early bipolar disorders, and early borderline personality disorders**

# **Supplement 2**

Content

[**Supplement 2** 1](#_Toc198553314)

[Search strategy and inclusion criteria 1](#_Toc198553315)

[Search log 1](#_Toc198553316)

[Two-Step full-text screening 3](#_Toc198553317)

[Data extraction 3](#_Toc198553318)

[Supplementary Table 1 Assessment of included reviews on early interventions in people with first episodes at increased risk of severe disease progression according to the AMSTAR-2 checklist 4](#_Toc198553319)

[Extraction and evaluation of individual studies 7](#_Toc198553320)

[Excluded studies: 15](#_Toc198553321)

[Reviews and meta-analysis 15](#_Toc198553322)

[Individual trials 18](#_Toc198553323)

[Supplementary Table 2 Summary of reasons for exclusion for the meta-analyses and reviews. 21](#_Toc198553324)

[Supplementary Table 3 Summary of reasons for exclusion for the single trials. 21](#_Toc198553325)

[Duplicate Check 22](#_Toc198553326)

[Supplemental Table 4 Study duplicates by review. 22](#_Toc198553327)

[Duplicate Check References 22](#_Toc198553328)

[**Supplemental Results** 25](#_Toc198553329)

[Early Psychosis 25](#_Toc198553330)

[Early Bipolar Disorders 27](#_Toc198553331)

[Individual studies 27](#_Toc198553332)

[**Supplemental References** 28](#_Toc198553333)

## Search strategy and inclusion criteria

## Search log

EBM Reviews - Cochrane Central Register of Controlled Trials <January 2023>

EBM Reviews - Cochrane Database of Systematic Reviews <2005 to January 12, 2023>

Embase <1974 to 2023 January 12>

Ovid MEDLINE(R) ALL <1946 to January 12, 2023>

APA PsycInfo <1806 to January Week 2 2023>

| Search number | Query | Results |
| --- | --- | --- |
| 1 | (sever* and mental* and disorder*):ti,ab,kw | 264331 |
| 2 | (sever* and mental* and illness):ti,ab,kw | 98844 |
| 3 | (severe and psychiatric and disorder*):ti,ab,kw | 62217 |
| 4 | (severe and mental and health and problems):ti,ab,kw | 17319 |
| 5 | (depression or (depressive and disorder*) or (severe and affective and disorder*)):ti,ab,kw | 1854332 |
| 6 | (schizophrenia or psychotic* or paranoid or schizophrenic* or psychosis) :ti,ab,kw | 782868 |
| 7 | ((bipolar and disorder*) or (bipolar and affective and disorder*) or (manic and depression) or (bipolar and depression)):ti,ab,kw | 212604 |
| 8 | ((personality and disorder*) or borderline personality disorder*):ti,ab,kw | 297086 |
| 9 | MeSH descriptor: schizophrenia/ | 399923 |
| 10 | MeSH descriptor: depression/ | 641829 |
| 11 | MeSH descriptor: bipolar disorder/ | 140468 |
| 12 | 1 or 2 or 3 or 4 or 5 or 6 or 7 or 8 or 9 or 10 or 11 | 2851465 |
| 13 | first-episode*:ti,ab,kw | 55529 |
| 14 | recent-onset:ti,ab,kw | 16864 |
| 15 | early-phase*:ti,ab,kw | 93079 |
| 16 | early-stage*:ti,ab,kw | 452553 |
| 17 | first-episode*:ti,ab,kw | 55529 |
| 18 | first-presentation*:ti,ab,kw | 13167 |
| 19 | ((randomized and controlled and trial) or (randomised and controlled and trial)):ti,ab,kw | 2552021 |
| 20 | (random* and allocation):ti,ab,kw | 207580 |
| 21 | (random* and (trial or study)):ti,ab,kw | 4506516 |
| 22 | (systematic and (review or search*)):ti,ab,kw | 995338 |
| 23 | ((meta and analysis) or metaanaly* or (meta and analy*) or scoping review):ti,ab,kw | 852025 |
| 24 | random allocation/ | 219714 |
| 25 | 19 or 20 or 21 or 22 or 23 or 24 | 5576233 |
| 26 | (early psycho-social intervention* or early intervention*):ti,ab,kw | 110855 |
| 27 | Early treatment*:ti,ab,kw | 65205 |
| 28 | (early intervention or service* or program*):ti,ab,kw | 5936606 |
| 29 | Early Psychosocial treatment:ti,ab,kw | 8 |
| 30 | psychosocial intervention:ti,ab,kw | 12154 |
| 31 | integrated treatment:ti,ab,kw | 5367 |
| 32 | (First episode program* or service*):ti,ab,kw | 2912142 |
| 33 | Early onset team:ti,ab,kw | 10 |
| 34 | 26 or 27 or 28 or 29 or 30 or 31 or 32 or 33 | 6009370 |
| 35 | 13 or 14 or 15 or 16 or 17 or 18 | 622447 |
| 36 | 12 and 35 | 59824 |
| 37 | 25 and 34 and 36 | 3681 |
| 38 | remove duplicates from 37 | 2284 |

## Two-Step full-text screening

A stepwise, hierarchical approach was employed to identify the evidence. First, relevant systematic reviews and meta-analyses were identified, and in the second step, randomized controlled trials not included in these reviews were selected.

This study included articles published in English and German that have full texts available.

Publications that included patients at high risk for having a first episode of any of the diseases mentioned above were not primarily considered. However, they could be included if their proportion was less than 50% of the total population. Studies employing a psychotherapeutic approach (e.g., cognitive behavioral therapy) were not included. Non-randomized controlled trials, cross-sectional studies, systematic reviews including non-randomized controlled trials and cross-sectional studies, qualitative studies, study protocols, case reports, and conference reports were excluded.

## Data extraction

A data extraction form was developed for systematic reviews and randomized controlled trials to capture relevant data from each review or study, including study identification (title, authors, and year of publication, country), sample characteristics (sample size, age, diagnosis, duration of illness), study characteristics (design, nature of intervention, control intervention, topics, length and intensity of intervention, and length of follow-up), and main findings.

We categorized the results into three key areas: (a) design: review versus primary study, (b) diagnosis: specific disease, and (c) intervention: coordinated, specific, and multiprofessional early intervention versus circumscribed, specific psychosocial intervention.

The presentation of results was based on the original literature. We reported adjusted or unadjusted odds ratios for categorical outcomes, including remission, response, and adverse events. We reported adjusted or unadjusted hazard ratios when available for survival-related measures, such as time to recurrence or relapse. Mean differences were noted for continuous measures to enhance their interpretability. When effect size differences were not reported, they were estimated (if possible). Studies with lower or higher risk of bias are highlighted in the text, while the remaining studies with intermediate or moderate risk of bias are described only in tables.

Data extraction forms for included reviews and meta-analyses are available from the authors in German.

## Supplementary Table 1 Assessment of included reviews on early interventions in people with first episodes at increased risk of severe disease progression according to the AMSTAR-2 checklist

Shea BJ, Reeves BC, Wells G, Thuku M, Hamel C, Moran J, Moher D, Tugwell P, Welch V, Kristjansson E, Henry DA. AMSTAR 2: a critical appraisal tool for systematic reviews that include randomised or non-randomised studies of healthcare interventions, or both. BMJ. 2017 Sep 21;358:j4008

| **Autor** | Ratheesh et al. 2023 | Frawley et al. 2021 | Camacho-  Gomez &  Castellvi 2020 | Puntis et al. 2020 | Correll et al. 2018 | Marshall et al.2011 | Alvarez-Jimenez et al. 2011 |
| --- | --- | --- | --- | --- | --- | --- | --- |
| **AMSTAR-Item** |  |  |  |  |  |  |  |
| 1. Did the research questions and inclusion criteria for the review include the components of PICO? | yes | yes | yes | yes | yes | yes | yes |
| 2. Did the report of the review contain an explicit statement that the review methods were established prior to the conduct of the review and did the report justify any significant deviations from the protocol? | yes | partial yes | partial yes | yes | yes | yes | yes |
| 3. Did the review authors explain their selection of the study designs for inclusion in the review? | no | no | no | yes | no | Yes | no |
| 4. Did the review authors use a comprehensive literature search strategy? | partial yes | partial yes | partial yes | yes | partial yes | Yes | partial yes |
| 5. Did the review authors perform study selection in duplicate? | yes | no | no | yes | yes | yes | yes |
| 6. Did the review authors perform data extraction in duplicate? | yes | yes | yes | yes | yes | yes | yes |
| 7. Did the review authors provide a list of excluded studies and justify the exclusions? | partial yes | partial yes | yes | yes | partial yes | yes | partial yes |
| 8. Did the review authors describe the included studies in adequate detail? | yes | yes | yes | yes | yes | yes | yes |
| 9. Did the review authors use a satisfactory technique for assessing the risk of bias (RoB) in individual studies that were included in the review? | yes | no | yes | yes | yes | yes | yes |
| 10. Did the review authors report on the sources of funding for the studies included in the review? | no | no | no | yes | Yes | yes | no |
| 11. If meta-analysis was performed did the review authors use appropriate methods for statistical combination of results? | No meta-analysis  conducted | yes | yes | yes | yes | yes | yes |
| 12. If meta-analysis was performed, did the review authors assess the potential impact of RoB in individual studies on the results of the meta-analysis or other evidence synthesis? | No meta-analysis  conducted | no | yes | no | Yes | no | no |
| 13. Did the review authors account for RoB in individual studies when interpreting/ discussing the results of the review? | yes | yes | yes | yes | yes | yes | yes |
| 14. Did the review authors provide a satisfactory explanation for, and discussion of, any heterogeneity observed in the results of the review? | yes | yes | yes | no | yes | yes | yes |
| 15. If they performed quantitative synthesis did the review authors carry out an adequate investigation of publication bias (small study bias) and discuss its likely impact on the results of the review? | No meta-analysis  conducted | yes | yes | yes | yes | yes | yes |
| 16. Did the review authors report any potential sources of conflict of interest, including any funding they received for conducting the review? | yes | yes | yes | yes | yes | yes | yes |
| **Assessment quality critical items (question: 1,4,7,9,11,13,15)** | moderate | low | moderate | moderate | high | high | moderate |

Extraction and evaluation of individual studies **(**Risk of Bias for Single Studies)

| Chanen et al. 2022  Effect of 3 Forms of Early Intervention for Young People With Borderline Personality Disorder The MOBY Randomized Clinical Trial | | | |
| --- | --- | --- | --- |
| Methods | Single-blind randomized controlled Trial  **Intervention group 1 (IG):** The Helping Young People Early (HYPE) combined with weekly cognitive analytic therapy (CAT)  **Intervention group 2 (IG):** HYPE combined with a weekly befriending psychotherapy control condition  **Intervention group 3 (IG):** a general youth mental health service (YMHS) model, combined with befriending  **Duration:** 18 month  **Patients:** People diagnosed with a borderline personality disorder (BPD) | | |
| Participants | **N=139**  - Key inclusion criteria were (1) age 15 to 25 years (inclusive) and (2) Structured Clinical Interview forDSM-IV-TRAxis II Disorders20 diagnosis of BPD (identical to DSM-5 criteria).  **Exclusion:**  - Key exclusion criteria were (1) Structured Clinical Interview Axis I Disorders Patient Edition diagnosis of psychotic disorder within the past 12 months; (2) lifetime diagnosis of a schizophrenia spectrum or bipolar I or II disorder; and (3) prior evidence-based treatment for BPD  **Age** (years): 19  **Duration of (untreated) illness:** not reported | | |
| Interventions | **IG** **1** **(N= 46):**  - The Helping Young People Early (HYPE) combined with weekly cognitive analytic therapy (CAT)  - Key elements HYPE include a CAT-based relational model (relational clinical care), integrated clinical casemanagement and general and acute psychiatric care, an explicitly collaborative approach, assertive engagement, outreach care in the community when needed, active inclusion of families, and a focus on functional recovery.  - CAT is a time-limited psychotherapy that focuses on understanding problematic self-management and interpersonal relationship patterns and the thoughts, emotions, and behavioral responses that result from these patterns  **IG 2 (N= 46):**  - HYPE combined with a weekly befriending psychotherapy control condition  - Befriending has been used for common factors of psychotherapy, including time in therapy, participant expectations, and the client-therapist relationship. Befriending consists of talking about neutral topics of interest to the participant, such as sport, music, social, and vocational activities, while avoidingor redirecting the participant from emotionally loaded topics, such as symptoms or interpersonal problems.  **IG 3 (N=47):**  - a general youth mental health service (YMHS) model, combined with befriending  - The YMHS model was implemented at Headspace by mental health clinicians who had expertise in young people (but who were not BPD treatment specialists), working within a multidisciplinary group practice setting. Key elements included a focus on engagement and young people–friendly practice, diagnosis, and treatment of mental disorders, clinical case management when needed, medical or psychiatric consultation when clinically indicated, and access to acute crisis care. | | |
| Outcomes | **Primary outcome measures:**  - The primary outcome was psychosocial functioning (Inventory of Interpersonal Problems Circumplex Version (IIP-C) and the Social Adjustment Scale Self-report (SAS-SR))  **Secondary outcome measures:**  - Secondary outcomes included DSM-IV-TR BPD features, suicidal ideation, suicide attempts and nonsuicidal self-injury, depression, substance use, and client treatment satisfaction | | |
| Results | - baseline till 18 month  **Primary outcome measures:**  **HYPE + CAT vs befriending**  - IIP-C: SE=−4.2 (11.4); t= −0.37; df (93.94) p= 0.72  - SAS-SR: SE=0.1 (0.1); t= −0.82; df (108.7) p= 0.42  **HYPE vs YMHS + befriending**  - IIP-C: SE=19.8 (10.8); t= 1.84; df (90.58) p= 0.07  - SAS-SR: SE=0.1 (0.1); t= 0.08; df (104.0) p= 0.94  **Secondary outcome measures:**  **HYPE + CAT vs befriending**  - BPDSI: SE=1.7 (3.1); t= 0.53; df (103.6) p= 0.6  - BSS: SE=-3.6 (2.2); t= −1.66; df (86.1) p= 0.1  - CESD-R: SE=1.3 (4.5); t= 0.28; df (105.1) p= 0.78  - MADRS: SE=-2.6 (3.4); t= −0.78; df (95.1) p= 0.44  - AUDIT: SE=−0.5 (1.8); t= −0.27; df (123.5) p= 0.79  - OTI:  Alcohol SE=1.2 (8.5); t= 1.39; df (87.37) p= 0.17  Tobacco SE=1.6 (2.0); t= 0.84; df (92.84) p= 0.4  Cannabis SE=2.2 (1.2); t= 1.88; df (61.59) p= 0.07  - CSQ: SE=0.2 (1.0); t= 0.2; df (105.72) p= 0.84  **HYPE vs YMHS + befriending**  - BPDSI: SE=0.3 (3.0); t= 0.11; df (98.3) p= 0.91  - BSS: SE=-2.6 (2.1); t= 1.25; df (84.42) p= 0.22  - CESD-R: SE=-0.8 (4.2); t= -0.18; df (101.09) p= 0.86  - MADRS: SE=-3.7 (3.2); t= 1.15; df (91.97) p= 0.26  - AUDIT: SE=−2.1 (1.7); t= 1.25; df (119.4) p= 0.22  - OTI:  Alcohol SE=-1.0 (.8); t= -1.24; df (82.6) p= 0.22  Tobacco SE=1.9 (1.9); t= -1.0; df (88.13) p= 0.32  Cannabis SE=-1.9 (1.2); t= -1.59; df (57.93) p= 0.12  - CSQ: SE=0.3 (0.9); t= 0.4; df (102.41) p= 0.69  AUDIT: Alcohol Use Disorders Identification Test; BPDSI: Borderline Personality Disorder Severity Index; BSS: Beck Scale for Suicide Ideation; CAT, cognitive analytic therapy; CESD-R, Center for Epidemiologic Studies Depression Scale; CSQ, Client Satisfaction Questionnaire; HYPE, Helping Young People Early; IIP-C, Inventory of Interpersonal Problems Circumplex Version; MADRS, Montgomery-Äsberg Depression Rating Scale; OTI, Opiate Treatment Index Section II; SAS-SR, Social Adjustment Scale Self-report; YMHS, youth mental health service. | | |
| Risk of bias | | | |
| *Bias* | ***Authors’ judgement***  ***Low – Unclear – high risk*** | | ***Support for judgement*** |
| Random sequence generation (selection bias) | Low risk |  | Using computer-generated randomization sequences, prepared by an independent statistician, participants were randomly assigned both to 1 treatment arm (in a 1:1:1 ratio) and  to a clinician. Permuted blocking was used, and participants were stratified by age (cut point of age 18 years), sex assigned at birth, and Center for Epidemiological Studies Depression Scale–Revised score (cut point of 37). |
| Allocation concealment (selection bias) | unclear risk |  | - |
| Blinding of participants and personnel (performance bias) | unclearrisk |  | Participants were informed of their treatment assignment by the trial coordinator. |
| Blinding of outcome assessment (detection bias) | low risk |  | Assessors were blind to treatment assignment and to the study design. |
| Incomplete outcome data (attrition bias) | low risk |  | -Retention rates did not differ significantly between the 3 groups at any time point. |
| Selective reporting (reporting bias) | low risk |  | All outcome measures were considered in the analysis. |
| Other bias  (adequate therapy method, treatment fidelity, supervision, training, personal, financial, or any other interests  producing bias) | low risk |  | - |

| Chien et al. 2015  A randomized controlled clinical trial of a nurse-led structured psychosocial intervention program for people with first-onset mental illness in psychiatric outpatient clinics | | | |
| --- | --- | --- | --- |
| Methods | **Single-blind, parallel group, randomized controlled trial**  **Intervention group (IG):** nurse-led structured psychosocial intervention program (PIP)  **Control group (CG):** usual care  **Duration:** 4 months, 12 months Follow-Up  **Patients:** people with first-onset mental illness | | |
| Participants | **N=180**, - aged 18–60 years, with capacity to provide in-formed consent, able to understand Cantonese/Mandarin, having a first-onset of mental illness (psychotic and mood disorders) within the past three months, newly referred to mental health care services, and presenting at least mild to moderate–severe levels of psychiatric symptoms  - this study was conducted in two regional general psychiatric outpatient clinics (OPDs) serving a population of approximately 800,000 (12% of the total population) in Hong Kong  **Excluded:**  Patients who were receiving other psychosocial interventions or-  ganized by the clinics or other health care organizations, or who were classified as the highest priority of psychiatric consultation and treatment  **Age** (years): about 25  **Duration of illness (months):** about 3 | | |
| Interventions | **IG** **(N=90):**  - PIP aimed to empower patients to manage their psychiatric symptoms by  enhancing their coping skills and improving their understanding of mental illness  - face-to-face individual meetings by one psychiatric advanced practice  Nurse (APN)  - eight 2-hour sessions held every two weeks  - five themes:(a) orientation, engaging  and understanding of mental health/illness, its related behaviors and community support resources; (b) working collaboratively and empowering patients using motivational interviewing approaches; (c) social and interpersonal skills training; (d)coping strategy enhancement for symptoms of mental illness and anxiety; and (e) reviewing sessions and establishing a realistic plan for future  **CG (N=90):**  - usual care | | |
| Outcomes | Patients were measured at baseline and at the endpoint (after 3 months).  **Primary outcome measures:**  - patients’ mental state (BPRS)  **Secondary outcome measures:**  - patients’ awareness of illness and insight into their needs for treatment (ITAQ)  - self perception (PSES)  - Quality of Life (WHOQoL-BREF)  - Hospitalization and default follow-up rates | | |
| Results | **measured at 12 months follow up**  **Primary outcome measures:**  BPRS  - MD=6.2, F(1,177)=12.3, p=0.001, ES:0,56  **Secondary outcome measures:**  ITAQ  - MD=5.4, F(1,177)=14.8, p= 0.001, ES: 0,60  PSES  - MD=20.1, F(1,177)=3.89, p= 0.09  WHOQoL-BREF  - MD=16.4, F(1,177)=14.2, p=0.001, ES: 0,42  Average length of hospitalizations  - MD=3.2, F(1,177)=11.4, p=0.01, ES:0.45  default follow-up rates  - MD=1.3, F(1,177)=2.01, p=0.14 | | |
| Risk of bias | | | |
| *Bias* | ***Authors’ judgement***  ***Low – Unclear – high risk*** | | ***Support for judgement*** |
| Random sequence generation (selection bias) | low risk |  | Consenting patients were then randomly selected by an independent research assistant (not involved in outcome measurements, intervention and data analysis) to participate in this  study using two sets of computer-generated random numbers(i.e.,one for each clinic). |
| Allocation concealment (selection bias) | unclear risk |  | -Due to the nature of the psychosocial intervention it was not possible to blind participants to their treatment allocation.  However, to avoid subject biases or potential contamination of treatment effects, the participants were asked not to disclose their  study participation to the clinic staff. The participant lists were also locked away and concealed from the clinic staff and researchers over the study period. |
| Blinding of participants and personnel (performance bias) | unclear risk |  | - Due to the nature of the psychosocial intervention it was not possible to blind participants to their treatment allocation. |
| Blinding of outcome assessment (detection bias) | low risk |  | - Assessment of patients’ outcomes was performed by a researcher (first author) blind to group allocation |
| Incomplete outcome data (attrition bias) | low risk |  | -Eighty-six of the PIP participants (95.6%) completed the program (i.e., an attendance of at least five sessions); three participants in the PIP (3.3%) and four in the usual care group (4.4%)  could not be contacted at 12-month follow-up. |
| Selective reporting (reporting bias) | low risk |  | All outcome measures were considered in the analysis. |
| Other bias  (adequate therapy method, treatment fidelity, supervision, training, personal, financial, or any other interests  producing bias) | low risk |  | - |

| Chien and Leung 2013  A controlled trial of a needs-based, nurse-led psychoeducation programme for Chinese patients with first-onset mental disorders: 6 month follow up | | | |
| --- | --- | --- | --- |
| Methods | **Single-blinded, randomized, controlled trial**  **Intervention group (IG):**  - individual psychoeducation programme (plus the routine psychiatric care)  **Control group (CG):**  - usual care  **Duration:**  - 6 months  **Patients:**  - newly triaged patients with moderately severe mental health problems at one psychiatric OPD (Outpatient Department) in Hong Kong | | |
| Participants | **N=96**, The inclusion criteria for these patients in the OPD were: ageing >= 18; ability to understand Cantonese/Mandarin; having the first onset of mental illness; and being a new contact for mental health-care services  **Excluded:** Those patients who were receiving other psychosocial interventions or upgraded to the highest priority of psychiatric consultation before intervention were excluded  **Age** (years): 26  **Duration of illness (years):** 1,4 months | | |
| Interventions | **IG** **(N=48):**  - needs-based psychoeducation programme (NPEP)  - the programme was based on psychoeducation and supportive programmes  - six biweekly, 1 h sessions (over 3 months)  - The programme consisted of six themes, including orientation and engaging and understanding mental health and illnesses; sleep hygiene and allaying anxiety; psychiatric treatments and medications; coping with health problems; interpersonal skills; and community support resources and future plan  **CG (N=48):** usual care | | |
| Outcomes | Assessment was performed before the intervention and after completion (6 month)  **Primary outcome measures:**  - Patients’ mental state (18-item BPRS),  - insights into illness and treatment (The Insight and Treatment Attitudes Questionnaire (ITAQ)),  - self-efficacy in managing difficult life situations (10-item Perceived Self-Efficacy Scale (PSS)),  - and their utilization of community services (modified Family Support Services Index (M-FSSI))  - Rehospitalization (Duration, Number) | | |
| Results | **Primary outcome measures:**  **BPRS**  - significant differences favouring IG (IG: Mean 3,0; SD 0,8; CG: Mean 4,8; SD 1,8; p < 0.001)  **ITAQ**  - significant differences favouring IG (IG: Mean 15,2; SD 1,8; CG: Mean 12,4; SD 2,3; p < 0.01)  **PSS**  - significant differences favouring IG (IG: Mean 27,5; SD 4,2; CG: Mean 15,0; SD 4,2; p < 0.001)  **M-FSSI**  - no significant differences between groups  **Rehospitalization (Duration)**  - significant differences favouring IG (IG: Mean 5,1; SD 3,8; CG: Mean 14,1; SD 5,6;p < 0.01)  **Rehospitalization (Number)**  - no significant differences between groups | | |
| Risk of bias | | | |
| *Bias* | ***Authors’ judgement***  ***Low – Unclear – high risk*** | | ***Support for judgement*** |
| Random sequence generation (selection bias) | low risk |  | - The participants were assigned randomly to either the usual psychiatric care (n = 48) or the NPEP (plus usual care; n = 48) by drawing a labelled card. |
| Allocation concealment (selection bias) | unclear risk |  | - |
| Blinding of participants and personnel (performance bias) | unclear risk |  | -The OPD staff were blind to the study group, and  the participants were asked to maintain the confidentiality of their study participation. |
| Blinding of outcome assessment (detection bias) | unclear risk |  | - |
| Incomplete outcome data (attrition bias) | low risk |  | - All randomized participants completed trial |
| Selective reporting (reporting bias) | low risk |  | All outcome measures were considered in the analysis. |
| Other bias  (adequate therapy method, treatment fidelity, supervision, training, personal, financial, or any other interests  producing bias) | low risk |  | - |

Risk of Bias Summary Table

|  | Random sequence generation (selection bias) | Allocation concealment (selection bias) | Blinding of participants and personnel (performance bias)* | Blinding of outcome assessment (detection bias) | Incomplete outcome data (attrition bias) | Selective reporting (reporting bias) | Other bias |
| --- | --- | --- | --- | --- | --- | --- | --- |
| Chanen et al. 2022 |  |  |  |  |  |  |  |
| Chien et al. 2015 |  |  |  |  |  |  |  |
| Chien et al. 2013 |  |  |  |  |  |  |  |

**Blinding: Es wurde hier, wenn eindeutig erkennbar und möglich, nur die Verblindung der Teilnehmenden bewertet, da keine Verblindung der Behandler:innen im Bereich psychosozialer Therapien möglich ist.*

## Excluded studies:

### Reviews and meta-analysis

1. Aceituno, David; Vera, Norha; Prina, A. Matthew; McCrone, Paul (2019): Cost-effectiveness of early intervention in psychosis: systematic review. In: *The British journal of psychiatry : the journal of mental science* 215 (1), S. 388–394. DOI: 10.1192/bjp.2018.298.

**Reason for exclusion:** research question.

1. Barlati, Stefano & De Peri, Luca & Deste, G. & Vita, Antonio. (2015). Non-pharmacological interventions in early schizophrenia: Focus on cognitive remediation. Journal of Psychopathology. 21. 1-12.

**Reason for exclusion:** all trials included in other review.

1. Behan C, Masterson S, Clarke M. Systematic review of the evidence for service models delivering early intervention in psychosis outside the stand-alone centre. Early Interv Psychiatry. 2017 Feb;11(1):3-13. doi: 10.1111/eip.12334. Epub 2016 Apr 8. PMID: 27061731.

**Reason for exclusion:** not a systematic review.

1. Catalan A, Richter A, Salazar de Pablo G, Vaquerizo-Serrano J, Mancebo G, Pedruzo B, Aymerich C, Solmi M, González-Torres MÁ, Gil P, McGuire P, Fusar-Poli P. Proportion and predictors of remission and recovery in first-episode psychosis: Systematic review and meta-analysis. Eur Psychiatry. 2021 Nov 3;64(1):e69. doi: 10.1192/j.eurpsy.2021.2246. PMID: 34730080; PMCID: PMC8668449.

**Reason for exclusion:** type of study designs included.

1. De Maio M, Graham P, Vaughan D, Haber L, Madonick S. Review of international early psychosis programmes and a model to overcome unique challenges to the treatment of early psychosis in the United States. Early Interv Psychiatry. 2015 Feb;9(1):1-11. doi: 10.1111/eip.12132. Epub 2014 Feb 27. PMID: 24576137.

**Reason for exclusion:** not a systematic review.

1. Dondé C, Jaffiol A, Khouri C, Pouchon A, Tamisier R, Lejoyeux M, d'Ortho MP, Polosan M, Geoffroy PA. Sleep disturbances in early clinical stages of psychotic and bipolar disorders: A meta-analysis. Aust N Z J Psychiatry. 2022 Sep;56(9):1068-1079. doi: 10.1177/00048674211068395. Epub 2021 Dec 31. PMID: 34971518.

**Reason for exclusion:** type of intervention

1. Hansen HG, Speyer H, Starzer M, Albert N, Hjorthøj C, Eplov LF, Nordentoft M. Clinical Recovery Among Individuals With a First-Episode Schizophrenia an Updated Systematic Review and Meta-Analysis. Schizophr Bull. 2023 Mar 15;49(2):297-308. doi: 10.1093/schbul/sbac103. PMID: 36029094; PMCID: PMC10016416.

**Reason for exclusion:** type of study designs included.

1. Harvey PO, Lepage M, Malla A. Benefits of enriched intervention compared with standard care for patients with recent-onset psychosis: a metaanalytic approach. Can J Psychiatry. 2007 Jul;52(7):464-72. doi: 10.1177/070674370705200709. PMID: 17688011.

**Reason for exclusion:** type of study designs included.

1. Howes OD, Whitehurst T, Shatalina E, Townsend L, Onwordi EC, Mak TLA, Arumuham A, O'Brien O, Lobo M, Vano L, Zahid U, Butler E, Osugo M. The clinical significance of duration of untreated psychosis: an umbrella review and random-effects meta-analysis. World Psychiatry. 2021 Feb;20(1):75-95. doi: 10.1002/wps.20822. PMID: 33432766; PMCID: PMC7801839.

**Reason for exclusion:** not a systematic review.

1. Hui TT, Garvey L, Olasoji M. Improving the physical health of young people with early psychosis with lifestyle interventions: Scoping review. Int J Ment Health Nurs. 2021 Dec;30(6):1498-1524. doi: 10.1111/inm.12922. Epub 2021 Aug 12. PMID: 34390119.

**Reason for exclusion:** not a systematic review.

1. Joyce K, Thompson A, Marwaha S. Is treatment for bipolar disorder more effective earlier in illness course? A comprehensive literature review. Int J Bipolar Disord. 2016 Dec;4(1):19. doi: 10.1186/s40345-016-0060-6. Epub 2016 Sep 9. PMID: 27613276; PMCID: PMC5017982.

**Reason for exclusion:** type of study designs included.

1. Killackey E, Yung AR. Effectiveness of early intervention in psychosis. Curr Opin Psychiatry. 2007 Mar;20(2):121-5. doi: 10.1097/YCO.0b013e328017f67d. PMID: 17278908.

**Reason for exclusion:** not a systematic review.

1. Lee R, Leighton SP, Thomas L, Gkoutos GV, Wood SJ, Fenton SH, Deligianni F, Cavanagh J, Mallikarjun PK. Prediction models in first-episode psychosis: systematic review and critical appraisal. Br J Psychiatry. 2022 Jan 24;220(Spec Iss 4 Themed Iss Precision Medicine and Personalised Healthcare in Psychiatry):1-13. doi: 10.1192/bjp.2021.219. Epub ahead of print. PMID: 35067242; PMCID: PMC7612705.

**Reason for exclusion:** research question.

1. Li Y, Coster S, Norman I, Chien WT, Qin J, Ling Tse M, Bressington D. Feasibility, acceptability, and preliminary effectiveness of mindfulness-based interventions for people with recent-onset psychosis: A systematic review. Early Interv Psychiatry. 2021 Feb;15(1):3-15. doi: 10.1111/eip.12929. Epub 2020 Feb 9. PMID: 32037721.

**Reason for exclusion:** type of study designs included.

1. Miley, Kathleen; Hadidi, Niloufar; Kaas, Merrie; Yu, Fang (2020): Cognitive Training and Remediation in First-Episode Psychosis: A Literature Review. In: *J Am Psychiatr Nurses Assoc* 26 (6), S. 542–554. DOI: 10.1177/1078390319877952.

**Reason for exclusion:** type of intervention.

1. Morriss RK, Faizal MA, Jones AP, Williamson PR, Bolton C, McCarthy JP. Interventions for helping people recognise early signs of recurrence in bipolar disorder. Cochrane Database Syst Rev. 2007 Jan 24;2007(1):CD004854. doi: 10.1002/14651858.CD004854.pub2. PMID: 17253526; PMCID: PMC6544804.

**Reason for exclusion:** type of intervention.

1. Müller, Hendrik & Bechdolf, Andreas. (2013). Psychologische Interventionen bei Personen mit erhöhtem Psychoserisiko. Psychotherapeut. 58. 10.1007/s00278-013-0996-2.

**Reason for exclusion:** Population.

1. Nordentoft M, Rasmussen JO, Melau M, Hjorthøj CR, Thorup AA. How successful are first episode programs? A review of the evidence for specialized assertive early intervention. Curr Opin Psychiatry. 2014 May;27(3):167-72. doi: 10.1097/YCO.0000000000000052. PMID: 24662959.

**Reason for exclusion:** not a systematic review.

1. Oliver D, Davies C, Crossland G, Lim S, Gifford G, McGuire P, Fusar-Poli P. Can We Reduce the Duration of Untreated Psychosis? A Systematic Review and Meta-Analysis of Controlled Interventional Studies. Schizophr Bull. 2018 Oct 17;44(6):1362-1372. doi: 10.1093/schbul/sbx166. PMID: 29373755; PMCID: PMC6192469.

**Reason for exclusion:** type of study designs included.

1. Rosenbaum S, Lederman O, Stubbs B, Vancampfort D, Stanton R, Ward PB. How can we increase physical activity and exercise among youth experiencing first-episode psychosis? A systematic review of intervention variables. Early Interv Psychiatry. 2016 Oct;10(5):435-40. doi: 10.1111/eip.12238. Epub 2015 Mar 23. PMID: 25808450.

**Reason for exclusion:** type of study designs included.

1. Santesteban-Echarri O, Piskulic D, Nyman RK, Addington J. Telehealth interventions for schizophrenia-spectrum disorders and clinical high-risk for psychosis individuals: A scoping review. J Telemed Telecare. 2020 Jan-Feb;26(1-2):14-20. doi: 10.1177/1357633X18794100. Epub 2018 Aug 22. PMID: 30134781.

**Reason for exclusion:** type of study designs included.

1. Shannon A, McGuire D, Brown E, O'Donoghue B. A systematic review of the effectiveness of group-based exercise interventions for individuals with first episode psychosis. Psychiatry Res. 2020 Nov;293:113402. doi: 10.1016/j.psychres.2020.113402. Epub 2020 Aug 19. PMID: 32862064.

**Reason for exclusion:** type of study designs included.

1. Shields, Gemma E.; Buck, Deborah; Varese, Filippo; Yung, Alison R.; Thompson, Andrew; Husain, Nusrat et al. (2022): A review of economic evaluations of health care for people at risk of psychosis and for first-episode psychosis. In: *BMC psychiatry* 22 (1), S. 126. DOI: 10.1186/s12888-022-03769-7.

**Reason for exclusion:** research question.

1. Sizer H, Brown E, Geros H, Yung A, Nelson B, McGorry P, O'Donoghue B. Outcomes for first-episode psychosis after entry via an at-risk mental state clinic compared to direct entry to a first episode of psychosis service: A systematic review and meta-analysis. Schizophr Res. 2022 Feb; 240:214-219. doi: 10.1016/j.schres.2021.12.019. Epub 2022 Jan 13. PMID: 35032907.

**Reason for exclusion:** type of study designs included.

### Individual trials

As a first step, primary studies were checked to see if they were included in the included reviews. Due to their abundance, these are not explicitly listed here.

1. Alvarez-Jiménez, Mario; Martínez-García, Obdulia; Pérez-Iglesias, Rocío; Ramírez, Mari Luz; Vázquez-Barquero, Jose Luis; Crespo-Facorro, Benedicto (2010): Prevention of antipsychotic-induced weight gain with early behavioural intervention in first-episode psychosis: 2-year results of a randomized controlled trial. In: *Schizophrenia research* 116 (1), S. 16–19. DOI: 10.1016/j.schres.2009.10.012.

**Reason for exclusion:** type of intervention.

1. Alvarez-Jimenez, Mario; Koval, Peter; Schmaal, Lianne; Bendall, Sarah; O'Sullivan, Shaunagh; Cagliarini, Daniela, et al. (2021): The Horizons project: a randomized controlled trial of a novel online social therapy to maintain treatment effects from specialist first-episode psychosis services. In: *World psychiatry : official journal of the World Psychiatric Association (WPA)* 20 (2), S. 233–243. DOI: 10.1002/wps.20858.

**Reason for exclusion:** type of intervention.

1. Chien, Wai Tong; Bressington, Daniel; Chan, Sally W. C. (2018): A Randomized Controlled Trial on Mutual Support Group Intervention for Families of People With Recent-Onset Psychosis: A Four-Year Follow-Up. In: *Frontiers in psychiatry* 9, S. 710. DOI: 10.3389/fpsyt.2018.00710.

**Reason for exclusion:** type of intervention.

1. Chien, Wai Tong; Ho, Long Kwan; Gray, Richard; Bressington, Daniel (2022): A randomized controlled trial of a peer-facilitated self-management program for people with recent-onset psychosis. In: *Schizophrenia research* 250, S. 22–30. DOI: 10.1016/j.schres.2022.09.028.

**Reason for exclusion:** type of intervention.

1. Curtis J, Watkins A, Rosenbaum S, Teasdale S, Kalucy M, Samaras K, Ward PB. Evaluating an individualized lifestyle and life skills intervention to prevent antipsychotic-induced weight gain in first-episode psychosis. Early Interv Psychiatry. 2016 Jun;10(3):267-76. doi: 10.1111/eip.12230. Epub 2015 Feb 26. PMID: 25721464.

**Reason for exclusion:** trial design.

1. Fisher, Emily; Wood, Stephen J.; Upthegrove, Rachel; Aldred, Sarah (2020): Designing a feasible exercise intervention in first-episode psychosis: Exercise quality, engagement and effect. In: *Psychiatry research* 286, S. 112840. DOI: 10.1016/j.psychres.2020.112840.

**Reason for exclusion:** type of intervention.

1. Gleeson, John & Chanen, Andrew & Cotton, Sue & Pearce, Tracey & Newman, Belinda & McCutcheon, Louise. (2012). Treating co-occurring first-episode psychosis and borderline personality: A pilot randomized controlled trial. Early intervention in psychiatry. 6. 21-9. 10.1111/j.1751-7893.2011.00306.x.

**Reason for exclusion:** participants <20

1. Gleeson, John F. M.; Cotton, Sue M.; Alvarez-Jimenez, Mario; Wade, Darryl; Gee, Donna; Crisp, Kingsley et al. (2013): A randomized controlled trial of relapse prevention therapy for first-episode psychosis patients: outcome at 30-month follow-up. In: *Schizophrenia bulletin* 39 (2), S. 436–448. DOI: 10.1093/schbul/sbr165.

**Reason for exclusion:** type of intervention.

1. Halverson TF, Meyer-Kalos PS, Perkins DO, Gaylord SA, Palsson OS, Nye L, et al. Enhancing stress reactivity and wellbeing in early schizophrenia: A randomized controlled trial of Integrated Coping Awareness Therapy (I-CAT). Schizophr Res. 2021 ;235:91-101. [doi.org/10.1016/j.schres.2021.07.022](https://doi.org/10.1016/j.schres.2021.07.022).

**Reason for exclusion:** type of intervention.

1. Killackey, Eóin; Allott, Kelly; Jackson, Henry J.; Scutella, Rosanna; Tseng, Yi-Ping; Borland, Jeff et al. (2019): Individual placement and support for vocational recovery in first-episode psychosis: randomised controlled trial. In: *The British journal of psychiatry : the journal of mental science* 214 (2), S. 76–82. DOI: 10.1192/bjp.2018.191.

**Reason for exclusion:** type of intervention.

1. Le TP, Ventura J, Ruiz-Yu B, McEwen SC, Subotnik KL, Nuechterlein KH. Treatment engagement in first-episode schizophrenia: Associations between intrinsic motivation and attendance during cognitive training and an aerobic exercise program. Schizophr Res. 2023 Jan;251:59-65. doi: 10.1016/j.schres.2022.12.018. Epub 2022 Dec 26. PMID: 36577235; PMCID: PMC10163954.

**Reason for exclusion:** measured outcomes.

1. Lovell, Karina; Wearden, Alison; Bradshaw, Tim; Tomenson, Barbara; Pedley, Rebecca; Davies, Linda M. et al. (2014): An exploratory randomized controlled study of a healthy living intervention in early intervention services for psychosis: the INTERvention to encourage ACTivity, improve diet, and reduce weight gain (INTERACT) study. In: *The Journal of clinical psychiatry* 75 (5), S. 498–505. DOI: 10.4088/JCP.13m08503.

**Reason for exclusion:** type of intervention.

1. Lowen C, Hodgekins J, Pugh K, Berry C, Fitzsimmons M, French P, Sacadura C, Birchwood M, Jackson C, Baggott E, Bernard M, Fowler D. Measuring adherence in social recovery therapy with people with first episode psychosis. Behav Cogn Psychother. 2020 Jan;48(1):82-90. doi: 10.1017/S1352465819000432. Epub 2019 Aug 5. PMID: 31379311.

**Reason for exclusion:** measured outcomes**.**

1. Nuechterlein, Keith H.; McEwen, Sarah C.; Ventura, Joseph; Subotnik, Kenneth L.; Turner, Luana R.; Boucher, Michael et al. (2022): Aerobic exercise enhances cognitive training effects in first-episode schizophrenia: randomized clinical trial demonstrates cognitive and functional gains. In: *Psychological medicine*, S. 1–11. DOI: 10.1017/S0033291722001696.

**Reason for exclusion:** type of intervention.

1. Nuechterlein, Keith H.; Subotnik, Kenneth L.; Ventura, Joseph; Turner, Luana R.; Gitlin, Michael J.; Gretchen-Doorly, Denise et al. (2020): Enhancing return to work or school after a first episode of schizophrenia: the UCLA RCT of Individual Placement and Support and Workplace Fundamentals Module training. In: *Psychological medicine* 50 (1), S. 20–28. DOI: 10.1017/S0033291718003860.

**Reason for exclusion:** type of intervention.

1. Palma, C.; Farriols, N.; Frías, A.; Cañete, J.; Gomis, O.; Fernández, M. et al. (2019): Randomized controlled trial of cognitive-motivational therapy program (PIPE) for the initial phase of schizophrenia: Maintenance of efficacy at 5-year follow up✰. In: *Psychiatry research* 273, S. 586–594. DOI: 10.1016/j.psychres.2019.01.084.

**Reason for exclusion:** type of intervention.

1. Rojnic Kuzman M, Bosnjak Kuharic D, Kekin I, Makaric P, Madzarac Z, Koricancic Makar A, Kudlek Mikulic S, Bajic Z, Bistrovic P, Bonacin D, Vogrinc Z. Effects of Long-Term Multimodal Psychosocial Treatment on Antipsychotic-Induced Metabolic Changes in Patients With First Episode Psychosis. Front Psychiatry. 2018 Oct 16;9:488. doi: 10.3389/fpsyt.2018.00488. PMID: 30386261; PMCID: PMC6198174.

**Reason for exclusion:** trial design.

1. Steare, Thomas; O'Hanlon, Puffin; Eskinazi, Michelle; Osborn, David; Lloyd-Evans, Brynmor; Jones, Rebecca et al. (2020): Smartphone-delivered self-management for first-episode psychosis: the ARIES feasibility randomised controlled trial. In: *BMJ open* 10 (8), e034927. DOI: 10.1136/bmjopen-2019-034927.

**Reason for exclusion:** type of intervention.

1. Tempier R, Balbuena L, Garety P, Craig TJ. Does assertive community outreach improve social support? Results from the Lambeth Study of early-episode psychosis. Psychiatr Serv. 2012 Mar;63(3):216-22. doi: 10.1176/appi.ps.20110013. PMID: 22388528.

**Reason for exclusion:** secondary analysis.

1. Ventura, Joseph; McEwen, Sarah; Subotnik, Kenneth L.; Hellemann, Gerhard S.; Ghadiali, Manali; Rahimdel, Amir et al. (2021): Changes in inflammation are related to depression and amount of aerobic exercise in first episode schizophrenia. In: *Early Interv Psychiatry* 15 (1), S. 213–216. DOI: 10.1111/eip.12946.

**Reason for exclusion:** type of intervention.

## Supplementary Table 2 Summary of reasons for exclusion for the meta-analyses and reviews.

| **Reason for exclusion** | **Meta-analyses and reviews count** |
| --- | --- |
| Type of study designs included | 10 |
| Not a systematic review | 6 |
| Research question | 3 |
| Type of intervention | 3 |
| All trials included in other review | 1 |
| Population | 1 |
| **Total** | **24** |

## Supplementary Table 3 Summary of reasons for exclusion for the single trials.

| **Reason for exclusion** | **Single trials count** |
| --- | --- |
| Type of intervention | 14 |
| Trial design | 2 |
| Measured outcomes | 2 |
| Participants <20 | 1 |
| Secondary analysis | 1 |
| **Total** | **20** |

## Duplicate Check

## Supplemental Table 4 Study duplicates by review.

| **Trials**  **Acronym/**  **Autor**  **Year** | **Frawley et al.**  **2021** | **Puntis et al.**  **2020** | **Camacho-**  **Gomez &**  **Castellvi 2020** | **Correll et al.,**  **2018** | **Marshall et al. 2011** | **Alvarez-**  **Jimenez et al.**  **2011** |
| --- | --- | --- | --- | --- | --- | --- |
| COAST |  |  |  | **X** |  |  |
| EASY_Extended |  | **X** |  |  |  |  |
| JCEP |  |  |  | **X** |  |  |
| Malla et al., 2017 |  | **X** |  |  |  |  |
| LEO |  |  |  | **X** |  | **X** |
| OPUS I |  |  | **X** |  | **X** | **X** |
| OPUS II |  | **X** |  |  |  |  |
| RAISE | **X** |  |  | **X** |  |  |
| STEP |  |  |  | **X** |  |  |

## Duplicate Check References

**COAST**

Kuipers E, Holloway F, Rabe-Hesketh S, Tennakoon L. An RCT of early intervention in psychosis: Croydon Outreach and Assertive Support Team (COAST). Social Psychiatry and Psychiatric Epidemiology. 2004;39:358-63.

**EASY_Extended**

Chan GH, Jim OT, AuYang CW, Hui CL, Wong GH, Lam MM, et al. Effects of extended case management on functioning in people with early psychosis-preliminary findings of the EASY3 randomised controlled study. Schizophrenia Research.

Chang WC, Chan GH, Jim OT, Lau ES, Hui CL, Chan SK, et al. Optimal duration of an early intervention programme for first-episode psychosis: randomised controlled trial. The British Journal of Psychiatry. 2015;206(6):492-500.

Chang WC, Chan HK, Jim TT, Wong HY, Hui LM, Chan KW, et al. Randomized controlled trial evaluating 1-year extended case management for first-episode psychosis patients discharged from EASY program in Hong Kong. Schizophrenia Bulletin. 2013.

Chang WC, Kwong VW, Chan GH, Jim OT, Lau ES, Hui CL, et al. Prediction of functional remission in first-episode psychosis: 12-month follow-up of the randomized-controlled trial on extended early intervention in Hong Kong. Schizophrenia Research. 2016;173(1-2):79-83.

Chang WC, Kwong VW, Chan GH, Jim OT, Lau ES, Hui CL, et al. Prediction of motivational impairment: 12-month follow-up of the randomized-controlled trial on extended early intervention for first-episode psychosis. European Psychiatry. 2017;41:37-41.

Chang WC, Kwong VW, Chan GH, Jim OT, Lau ES, Hui CL, et al. Sustainability of treatment effect of a 3-year early intervention programme for first-episode psychosis. The British Journal of Psychiatry. 2017;211(1):37-44.

Chang WC, Kwong VW, Or CF, Lau ES, Chan GH, Jim OT, et al. Motivational impairment predicts functional remission in first-episode psychosis: 3-Year follow-up of the randomized controlled trial on extended early intervention. Australian & New Zealand Journal of Psychiatry. 2018;52(12):1194-201.

Chen EY, Chang WC, Lee HM, Chan KW, Hui CL. Critical period in early psychosis intervention: possible dose effect from longitudinal studies from Hong Kong. Early Intervention in Psychiatry. 2016.

Chen EY, Chang WC, Chan SK, Lam MM, Hung SF, Chung DW, et al. Three-year community case management for early psychosis: a randomised controlled study. Hong Kong Medical Journal. 2015;21(Suppl 2):23-6.

Ho RW, Chang WC, Kwong VW, Lau ES, Chan GH, Jim OT, et al. Prediction of self-stigma in early psychosis: 3-year follow-up of the randomized-controlled trial on extended early intervention. Schizophrenia Research. 2018;195:463-8.

Kwong VW, Chang WC, Chan GH, Jim OT, Lau ES, Hui CL, et al. Clinical and treatment-related determinants of subjective quality of life in patients with first-episode psychosis. Psychiatry Research. 2017;249:39-45.

**JCEP**

Hui CL, Chang WC, Chan SK, Lee EH, Tam WW, Lai DC, et al. Early intervention and evaluation for adult-onset psychosis: the JCEP study rationale and design. Early Intervention in Psychiatry. 2014;8(3):261-8.

Hui CL, Lau WW, Leung CM, Chang WC, Tang JY, Wong GH, et al. Clinical and social correlates of duration of untreated psychosis among adult-onset psychosis in Hong Kong Chinese: the JCEP study. Early Intervention in Psychiatry. 2015;9(2):118-25.

**LEO**

Craig TK. The Lambeth Early Onset (LEO) Team: randomised controlled trial of the effectiveness of specialised care for early psychosis. BMJ. 2004;329(7474):1067.

Gafoor R, Nitsch D, McCrone P, Craig TK, Garety PA, Power P, et al. Effect of early intervention on 5-year outcome in non-affective psychosis. The British Journal of Psychiatry. 2010;196(5):372-6.

Garety PA, Craig TK, Dunn G, Fornells-Ambrojo M, Colbert S, Rahaman N, et al. Specialised care for early psychosis: symptoms, social functioning and patient satisfaction. The British Journal of Psychiatry. 2006;188(1):37-45.

McCrone P, Craig TK, Power P, Garety PA. Cost-effectiveness of an early intervention service for people with psychosis. The British Journal of Psychiatry. 2010;196(5):377-82.

Tempier R, Balbuena L, Garety P, Craig TJ. Does assertive community outreach improve social support? Results from the Lambeth Study of early-episode psychosis. Psychiatric Services. 2012;63(3):216-22.

**Malla 2017**

Albert N, Melau M, Jensen H, Hastrup LH, Hjorthøj C, Nordentoft M. T42. When should early intervention start, and for how long should it last? Schizophrenia Bulletin. 2018;44(Suppl 1):S129.

Lutgens D, Iyer S, Joober R, Brown TG, Norman R, Latimer E, et al. A five-year randomized parallel and blinded clinical trial of an extended specialized early intervention vs. regular care in the early phase of psychotic disorders: study protocol. BMC Psychiatry. 2015;15:1-9.

Lutgens D, Joober R, Iyer S, Lepage M, Norman R, Schmitz N, et al. Progress of negative symptoms over the initial 5 years of a first episode of psychosis. Psychological Medicine. 2019;49(1):66-74.

Malla A, Abadi S, Joober R, Latimer E, Schmitz N, Brown T, et al. A randomized controlled evaluation of “extended specialized early intervention service” vs. “regular care” for long-term management of early psychosis: a pilot study. Schizophrenia Research. 2010;117(2):115-6.

Malla A, Joober R, Iyer S, Norman R, Schmitz N, Brown T, et al. Comparing three-year extension of early intervention service to regular care following two years of early intervention service in first-episode psychosis: a randomized single blind clinical trial. World Psychiatry. 2017;16(3):278-86.

Malla A, Norman R, Iyer S, Joober R, Brown T, Schmitz N, et al. Extending specialized early intervention service from 2 to 5 years: A randomized controlled trial. Early Intervention in Psychiatry. 2012;6:36.

**OPUS I**

Jeppesen P, Petersen L, Thorup A, et al. Integrated treatment of first-episode psychosis: effect of treatment on family burden: OPUS trial. Br J Psychiatry Suppl. 2005;48:s85-s90.

Petersen L, Jeppesen P, Thorup A, et al. A randomised multicentre trial of integrated versus standard treatment for patients with a first episode of psychotic illness. BMJ. 2005;331:602.

Petersen L, Nordentoft M, Jeppesen P, et al. Improving 1-year outcome in first-episode psychosis: OPUS trial. Br J Psychiatry Suppl. 2005;48:s98-s103.

**OPUS II**

Albert N, Melau M, Jensen H, Emborg C, Jepsen JR, Fagerlund B, et al. Five years of specialised early intervention versus two years of specialised early intervention followed by three years of standard treatment for patients with a first episode psychosis: randomised, superiority, parallel group trial in Denmark (OPUS II). BMJ. 2017;356.

Melau M, Bertelsen M, Jeppesen P, Krarup G, Nordentoft M, Thorup A, et al. A randomised clinical trial of the effect of five-years versus two-years specialised assertive intervention for first episode psychosis: the OPUS-II trial. Schizophrenia Research. 2010;117(2):526.

**RAISE-ETP**

Kane JM, Robinson DG, Schooler NR, Mueser KT, Penn DL, Rosenheck RA, et al. Comprehensive versus usual community care for first-episode psychosis: 2-year outcomes from the NIMH RAISE Early Treatment Program. Am J Psychiatry. 2015 Oct.

Rosenheck RA, Leslie D, Sint K, Lin H, Robinson DG, Schooler NR, et al. Cost-effectiveness of comprehensive, integrated care for first episode psychosis in the NIMH RAISE Early Treatment Program. Schizophr Bull. 2016;42(4):896-906.

**STEP**

Srihari VH, Tek C, Kucukgoncu S, Phutane VH, Breitborde NJ, Pollard J, et al. First-Episode Services for Psychotic Disorders in the US Public Sector: A Pragmatic Randomized Controlled Trial. Psychiatric Services. 2015.

# **Supplemental Results**

## Early Psychosis

**Cochrane I:** Three RCTs (LEO, England; OPUS, Denmark; OTP, Sweden) and one cluster RCT (RAISE, USA) with 1,145 participants were included. There was no effect on employment status (RR 1.21 [95% CI 0.94 to 1.55]; p = 0.15; k = 2; n = 951). A clear difference was observed between SEI and TAU, favoring the SEI group, regarding treatment satisfaction (SMD 0.69 [95% CI 0.51 to 0.88]; p <0.00001; k = 2; n = 463; I2 = 0%; low-certainty evidence). Risks of bias were reported for various quality criteria. Overall, the evidence corresponds to levels of evidence 1a-1b.

**Cochrane II:** Overall, the evidence was rated as uncertain. No benefit or uncertain benefit was observed regarding remission, number of hospital admissions, duration of hospital treatment, symptom severity, or psychosocial functioning [1]. Overall, the evidence was rated as uncertain. No benefit or uncertain benefit was observed regarding remission, number of hospital admissions, duration of hospital treatment, symptom severity, or psychosocial functioning

**Correll et al., 2018:** The included studies investigated specific multi-modal treatment programs, all of which encompassed psychopharmacology, psychoeducation, and various psychosocial interventions, including (1) cognitive-behavioral therapy (CBT; 7 studies), family interventions (7 studies), vocational and educational counseling (5 studies), social skills training (5 studies), and crisis interventions (4 studies), all coordinated and integrated by a team. Adherence to the EIS intervention was assessed using rating scales in 6 of the studies. With an average study duration of approximately 16 months, benefits at the end of treatment were derived from all meta-analytic results favoring EIS. Participation in education and employment improved (RR: 1.13 [95% CI, 1.03-1.24]; p = .01; k=6), along with reductions in positive (SMD: -0.22 [95% CI, -0.32 to -0.11]; p < .001; k=10) and negative symptomatology (SMD: -0.28 [95% CI, -0.42 to -0.14]; P < .001; k=10). Quality of life also improved (SMD: 0.23; [95% CI 0.00-0.46]; p = .046; k=4). For example, participants in EIS compared to TAU had a 12.6% higher likelihood of attending school or being employed (NNT3: 17.8) and showed a 24% to 30% improvement in recovery rates (NNT: 13.9). The mean (SD) of low-risk ratings in the 7 areas of the Cochrane Collaboration's Risk of Bias Assessment Tool was 5.2 (0.9) (range, 4-7), indicating an overall low risk of bias. Analyses for potential publication bias were conducted for the various analyses, and additional analyses were performed to assess increased risks. Assessments of heterogeneity between studies (I2 statistic) were almost all below 50%, except for remission (I2: 68.9%), indicating low heterogeneity. Overall, the evidence corresponds to level 1a.

**Frawly et al., 2021:** A total of 31 studies with 2,811 participants were included, alongside other studies involving participants at clinical high risk for psychosis. The incorporation of participants experiencing early psychosis, as well as those at clinical high risk for psychosis, led to some heterogeneity among the included studies; most participants had at least one psychotic episode (Table 1). The review considered family-based therapy (k=3), supported employment (k=3), multi-modal psychosocial interventions (k=7), CBT for Psychosis (k=8), and cognitive remediation training (CRT, k=10), the latter being less relevant to this review. The control interventions varied significantly (Table 1). In the subgroup analyses, benefits were observed with longer intervention durations (> 6 months) compared to shorter durations (SMD = 0.397, [95% CI 0.149-0.645], p = 0.0029 vs. SMD = 0.251, [95% CI (0.088-0.415], p = 0.003). Similar findings were noted when comparing the number of sessions. The effects were less pronounced in studies utilizing an active control group. Community-based interventions appeared superior to clinic-based interventions (SMD = 0.376, 95% CI (0.129-0.623), p = 0.003 vs. SMD = 0.264, 95% CI (0.081-0.447), p = 0.005).

It is important to note the limitations of the quality assessment due to the use of a less common quality assessment scale [2], which does not allow evaluation of randomization and blinding variations. No evidence of significant publication bias was found for CBT, CRT, and psychosocial multi-component interventions, including all studies. However, the limited number of studies on supported employment and family-based intervention groups precludes reliable conclusions.

**Camacho-Gomez and Castellvi (2020)** present several lines of evidence suggesting that actively involving the families of patients with recent psychosis can significantly reduce the risk of relapse. Family interventions may enhance relatives' understanding of the illness and its potential impacts across various functional levels, help them recognize warning signs of relapse, and underscore the importance of early and continuous treatment. Eleven RCTs were included in the analysis. Definitions of a relapse varied: (a) hospitalization and (b) specific increases in symptoms in particular areas. Family interventions also demonstrated benefits in reducing psychotic symptoms (TAU, SMD = -0.68 [95% CI: -1.14 to -0.22], I² = 76 %, k = 6). However, there was substantial heterogeneity among the studies. The authors' analyses indicate that this heterogeneity was primarily influenced by whether the individual studies focused on multi-component therapy. The quality of the studies or the assessment instruments did not contribute to the observed heterogeneity. Patients' functioning was assessed using the Specific Levels of Functioning Scale (SLOF) and, in one study, with the GAF. Patients with a first episode exhibited a non-significant reduction in psychotic symptoms compared to those receiving TAU plus other active interventions (SMD = -0.27 [95% CI: -0.82 to 0.28]). According to AHRQ standards, four studies were rated good, four fair, and three poor [3]. Overall, this evidence falls within the 1a evidence level.

## Early Bipolar Disorders

Ratheesh et al. (2023) identified 25 studies that encompassed 16 randomized controlled trials (RCTs) (N = 2,212) and nine non-randomized studies (N = 17,714) investigating interventions for individuals in the early stages of bipolar disorder [4]. In addition to psychopharmacotherapy, the efficacy of psychotherapy, psychoeducation, and family-oriented approaches was also explored [4]. Two studies focusing on Family-focused therapy (FFT) demonstrated effects on secondary endpoints, including the duration of depressive episodes [5] and the severity of manic symptoms [6]. Another study investigated the effectiveness of specialized multimodal outpatient care (which included guideline-conforming pharmacological interventions and post-discharge group-based psychoeducation) versus TAU (routine outpatient psychiatric services) [7]. Participants reported greater satisfaction with the treatment compared to those receiving treatment as usual [8].

## Individual studies

Chanen et al.: Regardless of the group, there was an improvement in primary and secondary outcomes from baseline to the 12-month time point, except for the severity of substance use and participant satisfaction. The latter remained high across all time points.

# **Supplemental References**

[1] Puntis S, Minichino A, De Crescenzo F, Harrison R, Cipriani A, Lennox B. Specialised early intervention teams (extended time) for recent‐onset psychosis. Cochrane Database of Systematic Reviews. 2020a(11. Art. No.: CD013287). <https://doi.org/DOI>: 10.1002/14651858.CD013287.pub2.

[2] Rokita KI, Dauvermann MR, Donohoe G. Early life experiences and social cognition in major psychiatric disorders: A systematic review. European psychiatry. 2018;53:123-33.

[3] Camacho-Gomez M, Castellvi P. Effectiveness of family intervention for preventing relapse in first-episode psychosis until 24 months of follow-up: a systematic review with meta-analysis of randomized controlled trials. Schizophrenia Bulletin. 2020;46(1):98-109.

[4] Ratheesh A, Hett D, Ramain J, Wong E, Berk L, Conus P, et al. A systematic review of interventions in the early course of bipolar disorder I or II: a report of the International Society for Bipolar Disorders Taskforce on early intervention. International journal of bipolar disorders. 2023;11(1):1.

[5] Miklowitz DJ, Axelson DA, Birmaher B, George EL, Taylor DO, Schneck CD, et al. Family-focused treatment for adolescents with bipolar disorder: results of a 2-year randomized trial. Archives of general psychiatry. 2008;65(9):1053-61.

[6] Miklowitz DJ, Schneck CD, George EL, Taylor DO, Sugar CA, Birmaher B, et al. Pharmacotherapy and family-focused treatment for adolescents with bipolar I and II disorders: a 2-year randomized trial. American Journal of Psychiatry. 2014;171(6):658-67.

[7] Kessing LV, Hansen HV, Hvenegaard A, Christensen EM, Dam H, Gluud C, et al. Treatment in a specialised out-patient mood disorder clinic v. standard out-patient treatment in the early course of bipolar disorder: randomised clinical trial. The British journal of psychiatry. 2013;202(3):212-9.

[8] Colom F, Reinares M, Pacchiarotti I, Popovic D, Mazzarini L, Martínez-Arán A, et al. Has number of previous episodes any effect on response to group psychoeducation in bipolar patients? A 5-year follow-up post hoc analysis. Acta Neuropsychiatrica. 2010;22(2):50-3.

[9] Halverson TF, Meyer-Kalos PS, Perkins DO, Gaylord SA, Palsson OS, Nye L, et al. Enhancing stress reactivity and wellbeing in early schizophrenia: A randomized controlled trial of Integrated Coping Awareness Therapy (I-CAT). Schizophrenia Research. 2021;235:91-101.
